# Northern bottlenose whales in a pristine environment respond strongly to close and distant navy sonar signals

**DOI:** 10.1098/rspb.2018.2592

**Published:** 2019-03-20

**Authors:** Paul J. Wensveen, Saana Isojunno, Rune R. Hansen, Alexander M. von Benda-Beckmann, Lars Kleivane, Sander van IJsselmuide, Frans-Peter A. Lam, Petter H. Kvadsheim, Stacy L. DeRuiter, Charlotte Curé, Tomoko Narazaki, Peter L. Tyack, Patrick J. O. Miller

**Affiliations:** 1 Sea Mammal Research Unit, School of Biology, Scottish Oceans Institute, University of St Andrews, St Andrews, UK; 2 Faculty of Life and Environmental Sciences, University of Iceland, Reykjavik, Iceland; 3 Department of Biosciences, University of Oslo, Oslo, Norway; 4 Acoustics and Sonar Research Group, Netherlands Organisation for Applied Scientific Research (TNO), The Hague, The Netherlands; 5 LKARTS-Norway, Skutvik, Norway; 6 Defence Systems, Norwegian Defence Research Establishment, Horten, Norway; 7 Department of Mathematics and Statistics, Calvin College, Grand Rapids, MI, USA; 8 Cerema—Ifsttar, UMRAE, Laboratoire de Strasbourg, Strasbourg, France

**Keywords:** *Hyperoodon ampullatus*, military sonar, cetacean, marine mammal, disturbance

## Abstract

Impact assessments for sonar operations typically use received sound levels to predict behavioural disturbance in marine mammals. However, there are indications that cetaceans may learn to associate exposures from distant sound sources with lower perceived risk. To investigate the roles of source distance and received level in an area without frequent sonar activity, we conducted multi-scale controlled exposure experiments (*n* = 3) with 12 northern bottlenose whales near Jan Mayen, Norway. Animals were tagged with high-resolution archival tags (*n* = 1 per experiment) or medium-resolution satellite tags (*n* = 9 in total) and subsequently exposed to sonar. We also deployed bottom-moored recorders to acoustically monitor for whales in the exposed area. Tagged whales initiated avoidance of the sound source over a wide range of distances (0.8–28 km), with responses characteristic of beaked whales. Both onset and intensity of response were better predicted by received sound pressure level (SPL) than by source distance. Avoidance threshold SPLs estimated for each whale ranged from 117–126 dB re 1 µPa, comparable to those of other tagged beaked whales. In this pristine underwater acoustic environment, we found no indication that the source distances tested in our experiments modulated the behavioural effects of sonar, as has been suggested for locations where whales are frequently exposed to sonar.

## 1. Introduction

Marine mammals rely on sound for their survival and may therefore be affected by anthropogenic noise in their environment. Negative impacts of noise may include hearing loss [1], auditory masking [2], displacement [3] and disruption of important behaviours such as foraging and resting [4], with potential cumulative long-term population-level effects [5]. Recent studies on effects of anthropogenic noise within the marine environment have focused on the vulnerability of mammals to various disturbance sources, including naval sonar [6].

Several atypical mass strandings of predominantly beaked whales have occurred in close spatio-temporal proximity to sonar exercises [7]. Though the exact causal pathway remains unclear, the dominant hypothesis is that behavioural change was pivotal [8,9]. Observational studies using bottom-mounted hydrophone arrays on naval training ranges in the Bahamas [10] and Hawaii [11] reported reductions in detections of echolocation clicks indicating that Blainville's beaked whales (*Mesoplodon densirostris*) move away from multi-ship sonar exercises. Experimental studies found that two Blainville's beaked whales [10], two Cuvier's beaked whales (*Ziphius cavirostris* [12]) and an individual northern bottlenose whale (*Hyperoodon ampullatus* [13]) carrying archival tags called DTAGs exhibited strong responses, including cessation of feeding and avoidance, when experimentally exposed to sonar. The response of a tagged Baird's beaked whale (*Berardius bairdii*) was similar to that of the other three species, but abated more quickly [14]. These six tagged beaked whales were all close (less than 8 km) to the sound source during the experimental exposures, whereas reactions at longer distances were reported by an observational study involving 16 satellite-tagged Cuvier's beaked whales. That study found that dive behaviour, including deep-dive interval, tended to change during sonar exercises [15]. Effects were reported to be mediated by source distance and the type of sonar system used, with stronger responses to helicopter-dipping sonars having less predictable movement patterns compared to hull-mounted sonars [15].

Most of these studies on beaked whales were conducted on or near naval training ranges, where animals are regularly exposed to distant sonars. Animals are more likely to perceive infrequent and unpredictable sounds as a threat [16] and previous experience with the stimulus can influence the potential severity of the impact [6]. Therefore, we hypothesized that beaked whales on naval training ranges may have learned to associate distant, predictable sonars with a lower perceived risk, thereby altering their responsiveness to distant exposures. By contrast, beaked whales, and other cetaceans, in more pristine acoustic underwater environments may not have made this association.

We conducted controlled exposures of sonar signals to northern bottlenose whales to investigate the effects of source distance and received level on the onset and magnitude of behavioural responses in an area without frequent sonar activity. Our experimental design aimed to expose individuals to the specific range of received levels that has been associated with behavioural responses in beaked whales, but at radically different distances to the focal whale in contrasting close and distant exposure treatments. We conducted a limited number of ‘multi-scale’ controlled exposure experiments on this elusive species, each with one focal animal (always carrying a DTAG). To record behaviour across wider spatial and longer temporal scales in each experiment, passive acoustic monitoring (PAM) devices and satellite tags were used to observe non-focal animals at greater source distances.

## 2. Material and methods

### (a) Study area and subject animals

**Figure 1.**
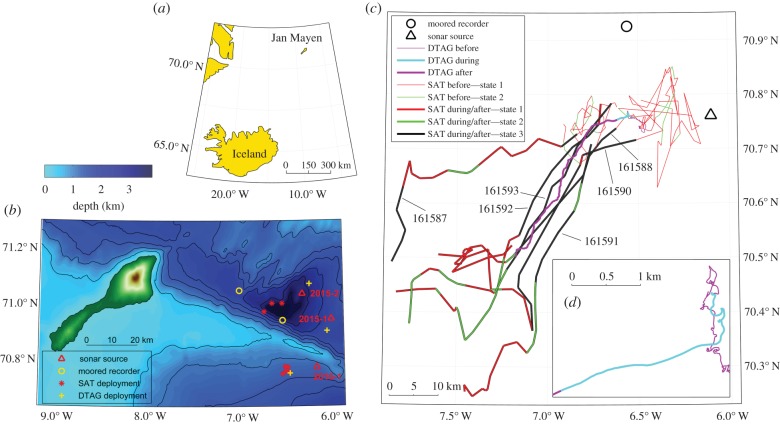
(*a*) Large-scale view. (*b*) Jan Mayen study area with all deployment locations (DTAG, focal whale; SAT, satellite-tagged whale). (*c*) Map of experiment 2016-1 with tracks of the tagged northern bottlenose whales before, during and up to 24 h after exposure. For satellite tags, colour-coding indicates the most likely sequence of states: state 1, tortuous movement; state 2, low-speed directional movement; and state 3, high-speed directional movement. (*d*) Detailed view of the track of the focal animal during exposure. Maps were created using the equidistant conic projection and GEBCO_2014 bathymetry data (www.gebco.net).

Fieldwork was conducted in June 2015 and 2016 in waters north of Iceland, near the remote island of Jan Mayen (Norway, 71° N–7° W) (figure 1*a*,*b*). Twelve northern bottlenose whales were instrumented with animal-borne tags (electronic supplementary material, table S1) and subsequently exposed to naval sonar signals in two experiments (2015-1 and 2015-2) with the close treatment and one experiment (2016-1) with the distant treatment. (A previous experiment with northern bottlenose whales, in June 2013, was also conducted in this area [13].)

### (b) Data collection

Data collection procedures, summarized here, are detailed in electronic supplementary material. We deployed two types of tags: short-term, high-resolution archival DTAGs [17] (*n* = 1 per experiment) that attached to a focal whale with suction cups, or medium-term, lower-resolution position and depth-transmitting satellite tags (experiment 2015-1: *n* = 0, 2015-2: *n* = 3, 2016-1: *n* = 6; electronic supplementary material, table S1). Satellite tags were programmed to continuously collect dive summaries and depth every 2.5 min for 1 day every 7 days. Dive summary profiles consisted of the start time and duration, maximum depth and shape (U, V or square) of each dive.

The base of operations was a 32 m motorized sailing vessel. A bottom-moored acoustic recorder (figure 1*b*) was deployed approximately 100 m above the seafloor to monitor for whale presence before, during and after exposure. For sound propagation modelling, we collected conductivity temperature depth (CTD) or expendable bathythermograph (XBT) measurements at locations between the source and tagged whale and near the mooring.

Each experimental cycle consisted of five phases: (i) searching, (ii) DTAG deployment, (iii) baseline pre-exposure, (iv) controlled exposure, and (v) post-exposure and DTAG recovery. We tried to satellite tag other groups in the study area throughout the fieldwork period, except during phases (iv) and (v). Once a DTAG was attached to a whale, this became the focal whale, which was tracked by observers visually and with a radio direction finder until the tag released from the animal. (Visual contact was temporarily lost during the distant experiment.) We attempted to satellite tag other individuals in the group for a maximum of 1 h after the DTAG was attached. Controlled exposure started after 4 h of baseline DTAG data were collected.

Each focal whale (with DTAG) was subjected to either a close or a distant exposure treatment. The source distance at the start of the exposure was less than 1 km (close) or 17 km (distant). In both treatments, we transmitted from the drifting sailing vessel a sequence of simulated sonar pulses that was representative of active sonars used by navies; however, the acoustic source and details of navigation protocol (e.g. positioning of the source, source depth) and transmission protocol (e.g. frequency band, signal type, ramp-up and exposure duration) differed between the treatments (table 1). These differences resulted predominantly from the two different source systems that were available to the study. Such minor differences in protocol were assumed to be negligible based upon the results from previous sonar behavioural response studies on beaked whales [10,12–14], which had comparable differences in source parameters but reported very similar response types and thresholds across studies.

**Table 1.** Transmission protocols used in the four experiments on northern bottlenose whales in the Jan Mayen area (this study and [12]). The 2013 experiment was part of the MD-based response intensity analysis, so its transmission protocol is provided as a reference.

									exposure duration			
experiment	treatment	frequency band (kHz)	signal type^a^	source level (dB re 1 µPa^2^ m^2^)	pulse duration (s)	pulse interval (s)	pulse onset (ms)	duty cycle (%)	ramp-up (min)	full- power (min)	source depth (m)	source movement
2015-1	close	1.0–2.0	tonal 1	∼122^b^	1	20	50	5	none	15	8	drifting
2015-2	close	1.0–2.0	tonal 1	185	1	20	50	5	none	15	8	drifting
2016-1	distant	3.4–3.9	tonal 2	154–214	1.5	25	12.5	6	20	15	17	drifting
2013 ([12])	intermediate^c^	1.0–2.0	tonal 1	152–214	1	20	50	5	20	15	90–100	sailing a box pattern

^a^Tonal 1 was a hyperbolic upsweep. Tonal 2 was a compound signal consisting of a 500 ms linear upsweep from 3350 to 3450 Hz followed by a 500 ms tone at 3600 Hz and a 500 ms tone at 3900 Hz.

^b^Stimuli were transmitted at a lower SL than intended due to amplifier malfunction.

^c^The source distance during this experiment ranged between 4.4 and 7.7 km (i.e. intermediate distances relative to the close and distant treatments).

### (c) Data processing

#### (i) DTAG time-series data

The following time-series variables (5 Hz sample rate) were extracted from the DTAG: (i) body orientation in terms of pitch, roll and heading, derived from acceleration and magnetic field strength [17]; (ii) depth, derived from pressure after correction for temperature effects [17]; (iii) speed-through-water for depths greater than 5 m, derived from acoustic flow noise in the 66–94 Hz band [18]; (iv) depth inflections—the proportion of zero crossings in the first difference time series of depth, calculated in a 30 s sliding window [13]; (v) circular variance in heading and circular variance in pitch, computed in a 1 min sliding window; (vi) pitching movement relative to the body axis for depths greater than 5 m; (vii) average overall dynamic body acceleration for depths greater than 5 m, computed with a 5 s averaging window.

#### (ii) Movement tracks for DTAGs and satellite tags

The horizontal track was estimated for each whale carrying a DTAG using track reconstruction [18]. This method estimated the animal's location at 1 s resolution by fitting a discrete-time correlated random walk model to the tag-derived displacement from dead-reckoning, and locations from visual sightings or Fastloc-GPS fixes. The tag recovery location was also used as an estimate of the location of the whale when the tag came off. An observation error s.d. of 10% was specified for the visual estimates of observer-whale range made from the crow's nest; all other parameter values were as in [18].

For each whale carrying a satellite tag, raw Argos locations were filtered using a random walk model fitted in a state-space framework in R package ‘crawl’ v. 2.1.1 [19] with modifications to incorporate error ellipse data [20]. Prior to model fitting, the raw Argos locations were passed through a speed filter (R package ‘argosfilter’ v. 0.63) with a threshold of 8 m s^−1^ to remove outliers. Analyses of the tracks were restricted to observations made between one week prior to the start of the sonar exposure and one week after its end. One model was fitted per whale with predictions of whale location (with uncertainty) made every 1 h.

#### (iii) DTAG audio recordings

DTAG audio files were inspected aurally and visually using spectrograms to identify the start and stop times of foraging sounds produced by the tagged whale, and those produced by other whales. Foraging sounds, consisting of echolocation search clicks and buzzes (which are likely to represent prey-capture attempts), were ascribed to the tagged whale depending upon the sounds' relative amplitude and spectral characteristics. Sonar signals were extracted from the audio files and received levels calculated following Miller *et al*. [4].

#### (iv) Received levels for satellite tags and animals near mooring locations

Satellite tags and bottom-moored recorders do not provide a measure of the received acoustic dose. To relate the acoustic dose of the sonar to the inferred behaviour, we modelled the received level of each transmitted sonar pulse with Bellhop [21]. Von Benda-Beckmann *et al*. [22] provide a detailed description of this analysis, summarized here. Propagation loss modelling was based upon sound speed measurements and the characteristic of the source (vertical beamwidth and in-beam source level (SL); table 1). All modelled SPLs were corrected for differences in averaging time (entire pulse versus 200 ms) based upon a comparison with SPLs measured from DTAG recordings. Normal distributions of depth uncertainty of the satellite tags (i.e. the differences between the sparser depth measurements and interpolations in the dive summary profile) were fitted to data. Separate distributions were fitted for animals at the surface and for animals that were diving. A Monte Carlo approach was then used to propagate forward the estimated depth uncertainty and horizontal positional uncertainty into the modelled received levels.

A similar approach was used for animals near the moored acoustic recorder location. However, as the exact location of the animals was unknown, we placed simulated animals at depths that were randomly drawn from pre-exposure satellite tag data and assumed an acoustic detection range of 1–4 km around the mooring location [22].

#### (v) Passive acoustic monitoring recordings

We scored the presence of northern bottlenose whale groups based upon acoustic detections of echolocation clicks during 2.5 min segments in two PAM recordings (electronic supplementary material, table S1). A band-limited energy detector using a guard band [23] was used; such a detector is well suited for the characteristic clicks of *H. ampullatus* [24]. Each time-bin was processed by applying a fast Fourier transform (FFT; length = 56.9 ms) using 50% overlapping segments and a Hanning window to estimate power spectral density (PSD). PSDs were normalized using the time average of the PSDs containing the lower 5% of the mean spectral levels in the 5–50 kHz band. Signal levels were estimated in the echolocation band (20–40 kHz) and guard band (4–8 kHz). Differences in level between these two bands were computed using the time average over the highest 1% of the normalized PSD spectrograms in the two bands. A detection of clicks was scored when this level difference passed a pre-defined detector threshold.

The automatic detector was tested and tuned using 416 manually audited snapshots (one 2.5 min segment every 45 min) taken from one PAM recording (JM1). Performance evaluation using receiver operating characteristic curves showed only limited dependency on the implemented percentiles and FFT length. An FFT length of 8192 points and the percentiles specified above were selected in combination with two detector thresholds: 5 dB (precision = 30%; recall = 17%) and 8 dB (precision = 25%; recall = 25%).

### (d) Evaluation of responses

We used four analytical methods to assess whether and how animals responded to the controlled exposures.

#### (i) Mahalanobis distance-based change-point analysis

We quantitatively compared behaviour of each focal whale (with DTAG) during and after exposure to its behaviour during a pre-exposure baseline period (from the first surfacing after the first deep dive until the start of the sonar exposure), by collapsing multivariate time-series data into a univariate time series of Mahalanobis distances (MDs). Each record was summarized by two MD metrics [13]; one designed for detecting changes in movement consistent with avoidance and one for tracking energetic cost of locomotion (variables the same as in [13]). For both sets of variables, we calculated the MD between the baseline-period average and the averages of 15 min windows centred at 1 min intervals [14]. A threshold criterion for change-point detection was derived by resampling 15 min windows from the baseline period 100 k times, and setting the threshold criterion at the 95th percentile of the maxima of the resampled periods. The 15 min averaging window was still too long to identify the start of the response precisely enough to match with a specific received SPL and distance. Therefore, the starts of the two movement responses were manually identified in the data by two panels of three authors (electronic supplementary material). Each panel independently identified the same start times.

#### (ii) Mahalanobis distance-based response intensity analysis

To investigate effects of source distance and SPLmax (the maximum SPL of the experiments) on a response intensity index, we calculated the MD between the baseline-period average and the averages of 35 min windows (the longest exposure duration) without overlap for the set of movement variables. Due to the limited number of experiments (*n* = 3), this analysis also included DTAG data from the published 2013 experiment (*n* = 1 exposed whale, intermediate source distance; electronic supplementary material, table S2) and from baseline tags (*n* = 10 whales) with representative natural behaviour collected near Jan Mayen in June 2013–2016 [13] (electronic supplementary material, figure S4). These MDs indicated how much each whale's movement behaviour within a time-bin deviated from the average baseline behaviour of all whales. For the four exposed whales, the start of the time-bins was aligned with the start of the exposure.

Following DeRuiter *et al*. [12], we modelled the response intensity index RI as

2.1 $$RI_{ik}=E(\mathrm{MD})=\left\{\begin{array}{cc} \beta_{0}+\frac{\beta_{1}L_{i}e^{\beta_{2}(\tau_{i}-t_{ik})}}{\left(1+\beta_{3}R_{i}\right)} & \mathrm{if}\;t_{ik}\geq \tau_{i}\\ \beta_{0} & \mathrm{otherwise} \end{array}\right.,$$

where RI is the expected value of MD, *i* = 1, … , 4 indexes the exposures, *k* indexes the time-bins of the exposed whales, *τ_i_* is the time-bin of exposure *i*, *L_i_* is the received SPLmax of exposure *i*, *R_i_* is the minimum source distance of exposure *i*, and *β*_0−3_ are four parameters that were estimated using maximum-likelihood estimation. Variable *L_i_* was offset by 79 dB re 1 µPa so that ‘no effect’ matched the hearing threshold of a beaked whale for a 5.6 kHz tone [24]. The observed MDs were modelled using a γ distribution, requiring estimation of an additional parameter (*ω*) related to the shape of the distribution [12]. The full model (equation (2.1)) and seven reduced models were fitted to the data and compared using the Akaike information criterion (AIC). For reduced models without the effect of received SPLmax, *β*_1_*L_i_* was replaced by a single parameter (*γ*).

#### (iii) State-based modelling for satellite tags

To investigate potential avoidance responses by the satellite-tagged whales, we fitted hidden Markov models using R package ‘MomentuHMM’ v. 1.4.2 [25] to the posterior mean locations of all filtered tracks. Models were built using hourly step lengths and turn angles, using Γ and von Mises distributions, respectively. Observations for time-steps without raw ARGOS locations were treated as missing data. The fitted models separated the data into tortuous movements (state 1), lower-speed directional movements (state 2) and high-speed directional movements (state 3). Models with three states were selected over models with two states based upon AIC and biological realism; models with four states did not converge. Based on the expectation that avoidance responses would involve switching out of the non-directional state, we modelled probabilities of transitioning from state 1 to each of the three states separately as function of covariates [26]. Models had either no covariates (baseline model), covariate time to recovery (from sonar exposure) or an interaction of time to recovery with received SPLmax or source distance. Time to recovery was constructed as a linear time decay with a maximum value of 8 h (determined using AIC) at the time-step of the sonar exposure. All individuals shared the same transition probability matrix. AIC and, for step lengths, residual plots, were used to determine and evaluate the best model, with the Viterbi algorithm used to predict the most likely state at each time-step. We defined the best model as the simplest model within ΔAIC < 2.

There were indications that four satellite-tagged whales in distant experiment 2016-1 were associated with some degree. Two tags were deployed on the same group during the same surfacing period and their horizontal tracks and, to a lesser degree, dive profiles, were sometimes correlated. Two other tags were deployed 6 h apart (electronic supplementary material, table S1) on individuals that were not visually confirmed to be in the same group, but their horizontal tracks (electronic supplementary material, figures S6 and S7) and dive profiles were also sometimes correlated. Therefore, we checked whether the results for all satellite-tagged whales were robust against non-independence of individuals by repeating the model selection procedure after omitting different combinations of one or two individuals from the dataset.

#### (iv) Analysis of click-absent periods for animals near the mooring location

The analysis of the PAM recordings aimed at detecting cessations of sound production, which is a common response of beaked whales to sonar [13]. Whale groups were considered sufficiently close to the recorder to determine whether a response occurred if at least one click-present period coincided with the last hour of the pre-exposure. If that was the case, we compared the duration of the last click-absent period that started during sonar exposure to the empirical cumulative distribution of the durations of click-absent periods observed during a control period specified as data recorded the same year prior to the first sonar experiment. Durations of click-absent periods were calculated from the output of the detector, although click-absent periods that started during a sonar experiment were also manually audited for the presence of fainter clicks that could be missed by the detector. Click-absent periods of less than or equal to 10 min were excluded from the baseline dataset as such gaps in clicking occur normally during foraging behaviour [13]. A threshold for response was set at the 95th percentile of the click-absent period durations.

## 3. Results

**Figure 2.**
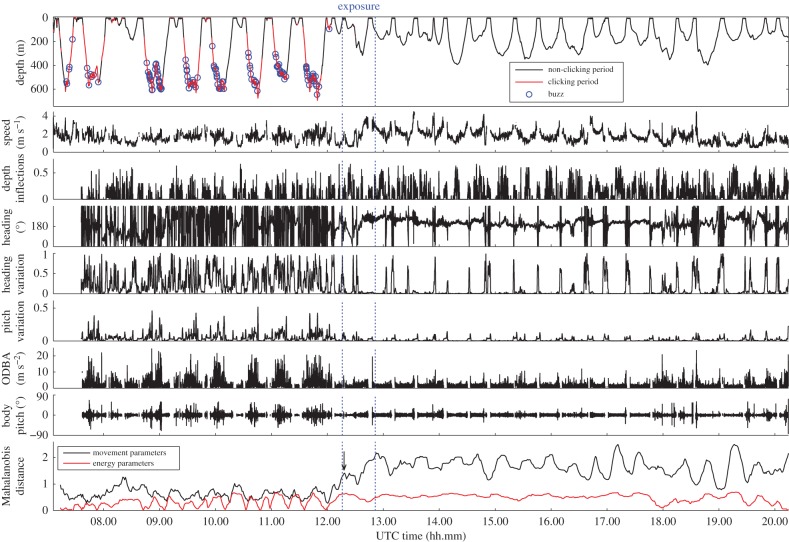
DTAG time-series data from a northern bottlenose whale that underwent controlled exposure to naval sonar during experiment 2016-1. The arrow indicates the middle of the first 15 min averaging window that reached the threshold criterion (i.e. the change-point) of the MD metric for avoidance movement.

**Figure 3.**
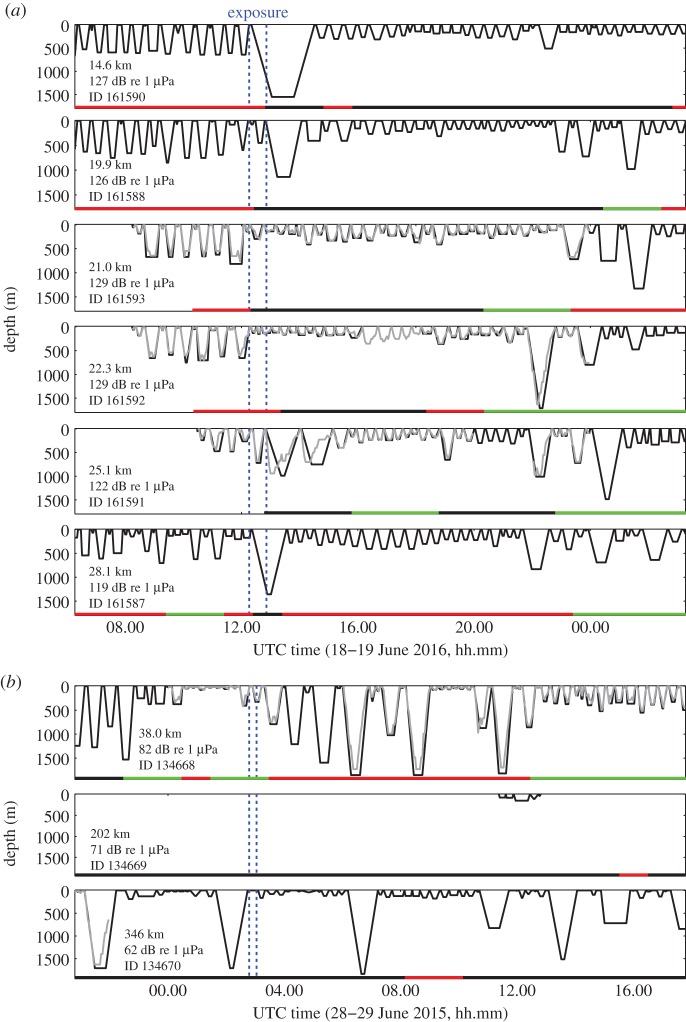
Dive summary (black) and regular depth (grey) profiles measured by satellite tags on northern bottlenose whales around the time of experiments (*a*) 2016-1 and (*b*) 2015-2. Panels are shown in order of mean distance during the exposure. Hourly state predictions based upon modelling of horizontal movement are indicated at the bottom of the panel, with the same colour-coding as in figure 1*c*. Note that most depth data were missing for ID134669. Also note the similarity in dive patterns between ID161588 and ID161590 and between ID161592 and ID161593, suggesting these whales were associated.

We conducted three experiments in 2015–2016 on a total of 12 tagged northern bottlenose whales (table 1; electronic supplementary material, table S1). Each experiment (2 close, 1 distant) included one focal whale carrying a DTAG. Two experiments included satellite-tagged whales, with *n* = 3 for close experiment 2015-2 and *n* = 6 for distant experiment 2016-1. Of those six, four whales (ID161588, ID161590, ID161592, ID161593), as well as focal whale ha16_170a, were associated with some degree based on visual observations and correlations in dive behaviour (figures 2 and 3*a*) and horizontal movement (figure 1*c*; electronic supplementary material, figures S6–S9). Short descriptions of the experiments are provided below (more detailed descriptions in the electronic supplementary material).

### (a) Close experiment 2015-1

Focal whale ha15_171a started foraging during the baseline period and continued for 2 h until the start of the exposure (electronic supplementary material, figure S1). Seconds after the first sonar signal (1.0–2.0 kHz, tonal 1; table 1) was received, the whale broke off a dive, ceased sound production and made a right turn towards the drifting source vessel. The whale started moving towards the source on a highly directed course and subsequently kept encircling it until the end of exposure. The first subsequent foraging dive started 24 min after the CEE had ended, suggesting that behavioural disruption due to this low-level sonar exposure (table 1) was relatively short. Change-points were not identified in the MD metrics for avoidance and change in locomotion (electronic supplementary material, figure S1).

### (b) Close experiment 2015-2

Focal whale ha15_179b made several deep foraging dives that were separated by shallow-diving bouts during baseline (electronic supplementary material, figure S2). Upon reception of the first sonar signal (1.0–2.0 kHz, tonal 1; table 1), the whale made a sudden movement and initiated a high-speed descent. Sounds from the whale were not recorded during this 840 m deep dive. The tag recorded elevated swim speeds, low variations in pitch and heading, and strong and consistent fluking throughout the exposure period. The whale kept moving away from the source location during and after exposure, for a total duration of 6.5 h. The change-point in the MD metric for avoidance was reached at the start of exposure, and these MDs remained elevated until the animal resumed foraging towards the end of the record (electronic supplementary material, figure S2). No change-point was identified in the MD metric for energetic cost of locomotion. Avoidance behaviour was not apparent for two satellite-tagged animals (ID134668, ID134670) and there were no observations during exposure for a third satellite-tagged animal (ID134669) (figure 3*b*). Clicks were not detected at the mooring location (26 km from the source) over a period between 6 h before and 4.8 h after exposure (electronic supplementary material, figure S3a).

### (c) Distant experiment 2016-1

During baseline, focal whale ha16_170a made regular foraging dives (figure 2) within a limited area (figure 1c). The exposure period (3.4–3.9 kHz, tonal 2; table 1) coincided with a dive that began as a typical shallow dive but then was extended in depth and duration (figure 2). Just before the final ascent, the animal started an avoidance response (figure 1*d*). Consistent clicking by the focal animal was not detected during or after exposure (figure 2). After the unusual dive, the animal kept moving away from the exposure site for longer than 7.5 h (figures 1*c* and 2). The tag was released 36.9 km from the location where the avoidance response had started. The change-point in the MD metric for avoidance was reached at the beginning of the exposure, and these MDs stayed elevated until the end of the record (figure 2). No change-point was identified in the MD metric for energetic cost of locomotion.

Six satellite tags were deployed (figure 1*c*), which included two tags (ID161592 and ID161593) on the same group as the focal animal. All six whales appeared to initiate avoidance responses, with animals travelling on directed courses for several hours after the exposure (figure 1*c*). Horizontal movements before exposure were classified predominantly as tortuous and thereafter mostly as high-speed directional (figure 1*c*). Four of the six whales initiated a long (1.2–2.2 h) and deep (992–1552 m) dive during exposure (figure 3*a*).

Northern bottlenose whale clicks were detected in the PAM recording during exposure, and these detections were followed by a 13.9 h click-absent period that started when the sonar was still transmitting (electronic supplementary material, figure S3b). This observation was a statistical outlier (at 0.05 level) compared with the durations of the pre-exposure click-absent periods (electronic supplementary material, figure S3c), suggesting that the exposure caused whale groups near the mooring location (25 km from the source) to stop echolocating and/or move out of the area. The received SPL for these groups at the start of the click-absent period was 95 dB re 1 µPa (electronic supplementary material, table S2).

### (d) Effect of received level or source distance

**Figure 4.**
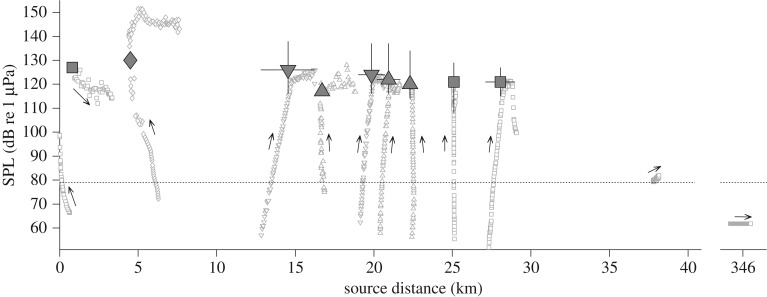
Avoidance response threshold SPLs (large symbols) for responses during sonar experiments versus source distance, for northern bottlenose whales carrying a DTAG (*n* = 4; less than 5 km and at 17 km) or a satellite tag (*n* = 8). Response thresholds were determined to be independent (filled square), from associated animals ID161588 and ID161590 (filled inverted triangle), associated animals ID161592, ID161593 and ha16_170a (filled triangle) or were from the 2013 experiment (filled diamond [12]). For satellite tags, the marker position indicates the maximum SPL and mean distance during exposure, vertical error bars a 90% confidence interval and horizontal error bars a min/max range. The data are detailed in electronic supplementary material, table S2. Small symbols indicate data for individual sonar pulses, with arrows showing their temporal progression. Whales can only respond if a signal is audible; therefore, the electrophysiological hearing threshold (dotted line) of another Ziphiid (Blainville's beaked whale [26]) is also shown.

The avoidance thresholds of the northern bottlenose whales exposed to sonar during the 2015–2016 experiments all were estimated to be within a narrow SPL range of 117–126 dB re 1 µPa but covered a wide range of source distances of 0.8–28 km (figure 4), suggesting that received level was a stronger driver of response onset than source distance. The lack of avoidance responses for three whales exposed to lower maximum SPLs (62–99 dB re 1 µPa) at distances of 0.02–346 km (figure 4) corroborate this conclusion. The narrow range of avoidance threshold SPLs was also consistent with the received levels predicted for whales near the bottom-moored acoustic recorders (electronic supplementary material, table S2).

For DTAG data including the three experiments described here, along with the 2013 experiment [13] (*n* = 4 exposed whales) and 10 baseline whales, the best model evaluating terms in equation (2.1) included an effect of received SPLmax but excluded effects of source distance and time since exposure (electronic supplementary material, table S3). The ΔAIC between the lowest ranked model including received level and the highest ranked model excluding received level was 55.3, providing strong support for an effect of SPLmax on response intensity. The model fit to the observed data was reasonable, although a small elevation during experiment 2015-1 was predicted that did not match the observations (electronic supplementary material, figure S4).

For satellite tag data (*n* = 9 whales), the model with the lowest AIC included an interaction between the effects of sonar (coded by the time to recovery from sonar exposure) and received SPLmax, but this model was not superior (ΔAIC < 2) to a model that included only effect of sonar (electronic supplementary material, table S4). There was more support for a sonar effect that only depended upon SPLmax than for one that only depended upon source distance (ΔAIC 10.6). These results were relatively robust against reduction in the dataset to account for potential non-independence of individuals (electronic supplementary material, table S5). However, ID161593 was particularly influential, and exclusion of this tag together with ID161588 reduced ΔAICs to less than 2. Predictions from the selected model indicated that the satellite-tagged whales' movements were less likely to remain tortuous and more likely to transition from tortuous to high-speed directional at the time of the exposure, compared to baseline (electronic supplementary material, figure S5).

## 4. Discussion

This study aimed to describe factors affecting responses of beaked whales to sonar in a remote area with little naval sonar activity; an area that can be considered acoustically pristine when compared with locations where similar studies have been conducted. During our experiments, the tagged whales exhibited behaviours that are characteristic for this species [13] and other Ziphiids (e.g. [10,12,28]), including sustained avoidance and cessation of feeding, at low received levels. Our results are based on a limited number of experiments (*n* = 3; *n* = 4 for the response intensity analysis), and thus, few exposure contexts and a limited total number of tagged individuals exposed to sonar. Nevertheless, the consistency in the thresholds and types of behavioural responses gives us confidence that these limited data provide novel information crucial to understanding effects of anthropogenic noise on beaked whales.

The estimated avoidance threshold SPLs (117–126 dB re 1 µPa) we identified for northern bottlenose whales were comparable to those previously measured for one conspecific (130 dB re 1 µPa [13]) and five other beaked whales (98–138 dB re 1 µPa [10,12,14]), but they were greater than 20 dB below the SPL associated with a 0.5 probability of response for Blainville's beaked whales during a multi-ship sonar exercise (150 dB re 1 µPa (SPLmax over 30 min windows) [28]). Due to the data resolution of the satellite tags and the lack of a ramp-up protocol during close experiments, the avoidance threshold SPLs of the six satellite-tagged whales and one whale carrying a DTAG (ha15_179b) represent an upper bound of the onset threshold SPL (figure 4). The step function for behavioural disturbance in beaked whales, used by the US Navy for environmental compliance [29], already reflects, to some degree, the heightened vulnerability of beaked whales to disturbance by noise from naval activities. However, our results indicate that a 140 dB re 1 µPa step function still underestimates behavioural disturbance to northern bottlenose whales in the off-sonar range context.

Tagging studies with Cuvier's beaked whales on or near a naval training range have reported that source distance may affect behavioural responses independent of received level. One Cuvier's beaked whale did not respond to incidental exposures from a distant (approx. 118 km) sonar at a received SPLmax of 106 dB re 1 µPa [12]. This SPL was 20 dB below the animal's onset threshold SPL measured during a close experimental exposure but exceeded that of a second animal [12]. Experimental exposure to high-power sonar from a distant (approx. 70 km) operational navy vessel also did not induce obvious behavioural reactions in another individual at received SPLs of 100–120 dB re 1 µPa [30]. Satellite tag deployments have also provided indications that source distance may mediate responsiveness. Changes in dive behaviour intensified with source proximity and were more pronounced in response to mid-power helicopter-deployed sonar exposure than in response to high-power ship-deployed sonar exposure at comparable distances within approximately 50 km, despite the lower SL of the mid-power sonar [15]. Beaked whales near naval training ranges may thus have learned to modulate their responsiveness based upon the perceived level of risk they associate with different source distances, SLs and/or source movements (i.e. the predictability of the exposures).

Here, source distance (to the 28 km tested) did not appear to influence responses. If the whales in our study associated more distant sources with lower perceived risk, then fewer responses with higher onset threshold SPLs would have been expected at greater distances. Those tagged whales that exhibited behavioural responses at longer distances were all part of the same experiment and in proximity to each other, suggesting that the behaviour of each whale may have influenced others in the experiment to some degree. Indeed, three whales were in the same social group when they were tagged, and the behaviour of these and two other whales around the time of the exposure was sometimes not independent of each other. However, the wide spacing of the tagged animals during exposure and the change in whale presence near the far-removed bottom-moored recorder (figure 1*c*) suggests that most groups responded independently (unless there was some unknown mechanism). The 2013 experiment off Jan Mayen also may have caused large-scale and sustained area avoidance in northern bottlenose whales [13]. The tagged whale's displacement in response to that experiment was greater than 33–36 km and coincided with a strong decline in acoustic and visual detection densities within a radius of approximately 10 km from the source (beyond which there was no recording effort). Data for northern bottlenose whales therefore do not support the hypothesis that distance modulates beaked whale responsiveness to sonar independent of received level. If bottlenose whales are not inherently more sensitive to disturbance by sonar than other beaked whales, the unpredictability of the exposures (due to the relatively pristine underwater acoustic habitat) could be the reason for the apparent contrast with beaked whale responses to ship-based sonar in areas with frequent sonar activity.

Cetaceans, and animals in general, might be more behaviourally responsive to anthropogenic noise in relatively pristine areas than in more industrialized areas. Belugas (*Delphinapterus leucas*) in the Canadian Arctic initiated avoidance responses to ice-breaking ships when the vessel noise was estimated to be barely audible to them, at 35–50 km [31,32]. This contrasts with belugas in an area with heavier shipping traffic, where the animals appear adapted to noisy vessels [33]. Other Arctic cetacean species have also been observed responding to anthropogenic noise at substantial distances from the source [31,32,34]. An important driver of the responses observed in this study may therefore have been the novelty of the stimulus in this environment, and not the type of stimulus *per se*.

Most tagged northern bottlenose whales that responded to sonar conducted a deep dive as a response, although some appeared to modify a shallow dive. The satellite-tagged whale (ID161590) that was closest to the source during experiment 2016-1 dove for a duration of 130 min, to a maximum depth that was close to the seafloor (figures 1 and 3). This dive duration may be a species record and is certainly feasible, since whalers have reported harpooned northern bottlenose whales diving for over 2 h [35] and since Cuvier's beaked whales exposed to sonar can dive for 163 min [15].

An important context of this study is that the underwater soundscape near Jan Mayen is largely pristine. A manual inspection of long-term averaged spectrograms of acoustic recordings over a 2-year period during, between and after the two field seasons confirmed that active sonar use is very uncommon in this area, with no naval sonar-like (1–10 kHz) signals identified in approximately 1500 h of recordings (S.P.v.IJ. 2018, unpublished data; electronic supplementary material). Some northern bottlenose whales migrate southwards through areas with more frequent sonar activity (e.g. the shelf edge region west of Scotland and Ireland [36]) and might hear sonar more regularly. We speculate that the whales might perceive the context of those sonar exposures as different due to the mismatch in time and space with the exposures near Jan Mayen.

Our approach of monitoring behaviour with complementary observational tools at different spatio-temporal scales during the same experiment was designed to maximize the amount of information per exposure. The approach limited the number of non-tagged individuals exposed in this pristine environment, and it allowed us to observe larger scale responses. The current multi-scale experimental design also has some important caveats, such as the reduced number of contexts in which animals are exposed, potential for non-independence of responses, difficulties in the identification of onset thresholds from lower-resolution data and a current lack of analysis methods to quantitatively integrate results from the different multi-scale sensors. Future studies using multi-scale study designs will require careful consideration of these issues. Here, the experimental design also included a transmission protocol which somewhat limited the interpretation of the data (e.g. close experiments were likely to produce left-censored response onset SPL thresholds). The preliminary evidence provided here should therefore be corroborated by information from additional experiments on northern bottlenose or other beaked whales in areas without frequent sonar activity. Such experiments should preferably expose individuals to received SPLs of 120–130 dB re 1 µPa at source distances greater than the maximum distance of 28 km that was tested here, to understand the full extent of habitat disruption that might be caused by operational naval sonars. This could probably only be achieved by using full-scale sonar sources (i.e. naval ships) during experimental studies, or as observational studies during actual naval exercises. To maximize the outcome and to minimize the number of exposures needed, we recommend the multi-scale approach demonstrated here, combining tags of different resolutions with other sensors such as moored or autonomous passive acoustic sensors.

## Supplementary Material

Supplementary text, figures and tables

## Data accessibility

All relevant data and scripts are within the paper and its electronic supplementary material, or available from the Dryad Digital Repository: https://doi.org/10.5061/dryad.fc5c0s4 [37].
