# Supplementary material for: Northern bottlenose whales in a pristine environment respond strongly to close and distant navy sonar signals
Source: Proc Biol Sci. 2019 Mar 20;286(1899):20182592. doi: 10.1098/rspb.2018.2592 (PMC6452067; doi:10.1098/rspb.2018.2592)
Supplement: Supplementary text, figures and tables [file rspb20182592supp1.pdf]

Electronic supplementary materials

For the manuscript:

Northern bottlenose whales in a pristine environment respond strongly to close and distant navy sonar signals

Paul J. Wensveen, Saana Isojunno, Rune R. Hansen, Alexander M. von Benda-Beckmann, Lars Kleivane, Sander van IJsselmuide, Frans-Peter A. Lam, Petter H. Kvadsheim, Stacy L. DeRuiter, Charlotte Curé, Tomoko Narazaki, Peter L. Tyack, Patrick J.O. Miller

Proceedings of the Royal Society B: Biological Sciences. doi:10.1098/rspb.2018.2592

Contents

Detailed methods - data collection 1

Detailed descriptions of the experiments 3

Long-term averaged spectrograms 8

Supplementary figures 9

Supplementary tables 16

Appendix I 21

Detailed methods - data collection

We deployed two types of tags: 1) short-term, high-resolution archival tags (DTAG; Johnson and Tyack, 2003) that attached to a focal whale with suction cups, and 2) medium-term, lower-resolution position and depth-transmitting satellite tags (SPLASH10, Wildlife Computers Inc.) in the low impact minimally percutaneous external-electronics transmitter (LIMPET) configuration (Andrews et al. 2008) (Table S1). Three versions of DTAG were used: version-2 and version-3 in their original configurations with VHF transmitter, and a ruggedized 'mixed-tag' with the DTAG version-3's sensor package in a modified housing that also contained a Fastloc-GPS logger (F3G 133A, Sirtrack Ltd.) and an Argos transmitter (SPOT-258E, Wildlife Computers Inc.). These additional sensors helped reconstruction of the whale track and aided retrieval of the tag once it had released. DTAGs recorded sound in stereo (version-2: 192 kHz; version-3: 240 kHz) as well as pressure, temperature, and triaxial acceleration and magnetic field strength (decimated to 5 Hz for analysis).

The base of operations was the 32-m motorised sailing vessel Donna Wood. The vessel had four VHF antennas mounted on top of its 25-m foremast that were connected to a radio direction finder (DFHorten, ASJ Electronic Design) to enable tracking of the focal animal at the surface. DTAGs were deployed from the deck using a pneumatic launcher (Aerial Rocket Tag System (ARTS), LK-ARTS) or from the bowsprit using a 7-m carbon fibre pole. Satellite tags were deployed from the deck of the sailing vessel or from a dinghy, using a pneumatic launcher (JM Standard, Dan-Inject ApS; or ARTS). Each year, an acoustic recorder (DSG-ST, Loggerhead Instruments Inc.) attached to a mooring was deployed upon arrival in the study area, which recorded continuous sound (144 kHz; –168 dB re V/μPa) before, during and after the experiments. At the end of each field season, this passive acoustic monitoring (PAM) device was redeployed with a reduced duty cycle of 6% (2.5/45 min) to allow for acoustic data collection until the next summer. The recorder was positioned at a depth of ~100 m above the sea floor, in a geographical location where bottlenose whale abundance was expected to be high (Fig. 1b). To enable accurate estimations of the sonar received levels experienced by the whales, we collected oceanographic measurements at locations between the source and the tagged whale and near the mooring using a conductivity, temperature, and depth-profiler (Mini-CTD, Valeport Ltd.) or an expendable bathythermograph (MK21/USB, Sippican Inc.).

The tagged whales were subsequently exposed to simulated naval sonar from the drifting sailing vessel. Once a DTAG was attached to a whale, this became the focal whales, which was tracked visually by observers on deck or in a 13-m high crow’s nest until the tag released from the animal. (Visual contact was temporarily lost for the Distant experiment). Surface observations collected by the visual observers at every surfacing period included the distance and bearing to the focal animal, and the size and composition of its group. Locations of the animals with satellite tags were tracked via the ARGOS system. Bottlenose whales are inquisitive animals and often approach boats, which is the best opportunity to place tags upon them. Therefore, we attempted to satellite tag other individuals in the group for a maximum of 1 h after the DTAG was attached. Controlled exposure started after 4 h of baseline DTAG data were collected.

Each focal whale (with DTAG) was subjected to either a Close exposure or a Distant exposure treatment. In both treatments we transmitted a sequence of simulated sonar pulses that was representative of signals from active sonars used by navies for long-range detection of targets. The acoustic source and details of navigation and transmission protocols differed between the experiments (Table 1). During Close exposure, the vessel was positioned to the front and side of the focal animal's travel path at the start of the experiment, at ~800 m from the animal. Sonar stimuli were amplified (Z8000, Cadence Sound Systems, Inc.) and projected into the water by a moving-coil transducer (LL9642T, Lubell Labs Inc.) at 8 m depth. During Distant exposure, the sailing vessel moved away from the focal animal to a location that was determined to maximise the SPL in the area where the animal was last sighted based upon *in-situ* acoustic propagation modelling. To produce the same target received SPLs as during Close exposure, the Distant exposure was performed using an underwater acoustic source system (ULHPAS, Applied Physical Sciences Corp.). This system consisted of a vertical line array of 15 disk transducers, a deck-top control unit, and a laptop. The source array was positioned at a mid-array depth of 17 m during transmission.

Tagging and experiments were conducted under permits from the Norwegian Animal Research Authority (permit no 2011/38782 and 2015/23222) and Icelandic Ministry of Fisheries in compliance with ethical use of animals in experimentation. Experimental procedures were also approved by the Animal Welfare Ethics Committee at the University of St Andrews.

Detailed descriptions of the experiments

The behaviour of the animals during each experiment was described in detail based on the DTAG, PAM and satellite tag data. These detailed descriptions, which can be found below, were partly based on expert-identification of the responses in the DTAG data (*sensu* Southall et al. 2007 and Miller et al. 2012) in addition to the other types of analyses. We used this method to judge whether a behavioural change had occurred, whether it was likely in response to the sonar, when the response started and when it approximately ended. Responses were first independently identified by two panels of four experts based upon inspection of standardised plots (Appendix I). One panel included authors C.C.,

S.I., and P.W. and the other panel authors P.K., F.P.L. and R.H. The panels were blind to each other's assessments, but were not blind to the experimental condition or timing of the exposure phase because they were familiar with the experiments. Thereafter, the two panels compared and assimilated their results in the presence of an adjudicator (author P.M.) to reach consensus (Table S6). Prior to consensus, 71% of responses were identified by both panels.

Close experiment 2015-1

When the group of whales was first encountered it consisted of 4 animals that were often getting close to ('seeking') the sailing vessel and 3-4 animals that stayed further away. All whales in the group were judged to be small adults or large juveniles. One animal in the group was tagged with a DTAG (ha15_171a). Group size remained 6-8 throughout the tracking period. The tagged whale started foraging about halfway into the baseline period, making deep dives (>452 m) with regular search clicks and foraging buzzes, and continued foraging for 2 h until the start of exposure (Fig. S1). The experiment started at 20-Jun-2015 15:13:00 and had a duration of 15 min. A few seconds after the first sonar signal was received, the whale broke off a foraging dive, ceased sound production, and made a right turn towards the drifting source vessel (66 dB re 1µPa; 633 m). Next, the whale started moving towards the source on a highly directed course and subsequently kept encircling the source vessel until the end of exposure, reaching a minimum distance of ~20 m. The first subsequent foraging dive of the animal started 24 min after the CEE had ended, suggesting that the behavioural disruption due to this low-level sonar exposure was relatively short. Change-points were not identified in the MD metric for avoidance or for change in locomotion (Fig. S1).

Bottlenose whale sounds were not detected in the PAM recording in the last 1.3 h before and during exposure (Fig. S3a), at 37 km from the source. However, click-present periods were frequently observed before that period and in the next two days, with the first one starting only 7.5 min after exposure (Fig. S3a), which suggested regular occurrence of northern bottlenose whales in the area. The sonar signals were likely inaudible to the animals close to the moored acoustic recorder location due to the combination of high transmission loss and low SL (Table 1).

Close experiment 2015-2

The whale was part of a group with 2 other small adults and one juvenile when it was tagged with a DTAG (ha15_179b). Before exposure, the whale made several deep foraging dives (>393 m) that were separated by bouts of one or more shallow dives (100-200 m) (Fig. S2). The sonar transmission sequence started at 29-Jun-2015 02:48:00 UTC and had a duration of 15 min (Table 1). The sonar exposure started when the whale was in a shallow diving bout. Upon reception of the first sonar signal, the whale made a sudden movement (potential startle response) and initiated a high-speed descent (127 dB re 1µPa; 814 m). Sounds from the focal whale were not recorded during the entire 840-m dive, and this lasted substantially beyond the end of exposure. The tag record showed highly elevated swim speeds, low variations in pitch and heading, and strong and consistent fluking motions throughout the exposure period. The whale's horizontal movement was directed away from the source location during and after exposure, for a total duration of 6.5 h, although the animal had started travelling on a course away from the source prior to exposure. Deep foraging dives were recorded again before the end of the tag record. A change-point in the MD metric for avoidance was reached in the beginning of the exposure period (but not for changes in locomotion), and the MD remained elevated until the animal resumed foraging (Fig. S2).

Three satellite tags were deployed prior to this experiment (Fig. 1b). The presence of a period of tortuous horizontal movements and deep dives indicated that one satellite-tagged whale (ID134668) initiated a 9-h foraging bout around the time of exposure (Fig. 3b). Avoidance behaviour was not apparent for this whale (82 dB re 1µPa; 38 km) (Fig. S12). The other two satellite-tagged whales were further from the sound source (>201 km) when the sonar transmissions started, because these two animals had been on a highly directed southern course for several days (Figs S13-S14). Their horizontal movements directly before and during exposure were classified as high-speed directional, hampering our ability to assess avoidance. However, the diving behaviour of one whale (ID134670) was undisturbed (62 dB re 1µPa; 345 km) so this whale did not appear to exhibit a strong avoidance

response (Fig. 3b). Dive data were not sufficiently available for the other whale (ID134669) to make the assessment (Figs 3b, 4; Table S2).

Bottlenose whale clicks were not detected at the moored acoustic recorder location (26 km from the source) over a period between 6 h before and 4.8 h after exposure (Fig. S3a), suggesting northern bottlenose whales were never close to the recorder for that period.

Distant experiment 2016-1

One DTAG (ha16_170a) was deployed on a group of 4 animals (1 immature male, 2 immature females, 1 subadult) prior to the experiment. During the 5.2-h baseline period the tagged whale made regular foraging dives to 500-700 m (Fig. 2) within a limited spatial area (Fig. 1c). The sonar transmission sequence, with a duration of 35 min, was initiated at 18-Jun-2016 12:16:00 UTC. This exposure period coincided with a 25-min dive (Fig. 2) that began as a typical shallow dive but then was extended in depth and duration (similar as in experiment 2015-2, Fig S2). The first response was judged to have occurred at the start of the first ascent period, when the animal simultaneously reduced speed and initiated a 360° turn in heading (77 dB re 1 µPa; 16.8 km). After several depth inflections and just before the final ascent, the animal started an avoidance response (117 dB re 1 µPa; 16.6 km) (Fig. 1d). Foraging buzzes or consistent periods of regular clicking by the focal animal were not detected during or after exposure (Fig. 2). After the unusual dive the animal kept moving away from the exposure site for >7.5 h (Figs 1c,2). The tag was released 36.9 km from the location where the avoidance response had started (in comparison, the horizontal displacement during the preceding 5.5 h had been only 3.5 km).

A change-point in the MD metric for avoidance movement was detected at the beginning of the exposure period, and these MDs stayed elevated until the end of the record (Fig. 2). No change-point was identified in the MD metric for energetic cost of locomotion.

Six satellite tags were deployed (Fig. 1c), which included two tags (ID161592 and ID161593) on members of the same group as the focal animal with DTAG. For these six whales, the source distance

at the start of exposure ranged from 12.8-27.1 km and received SPLmax from 120-126 dB re 1 µPa. All satellite-tagged whales appeared to initiate avoidance responses similar to the focal whale, with animals travelling on directed courses towards southwest for several hours after the exposure (Fig. 1c). Horizontal movements before exposure were classified predominantly as tortuous and thereafter mostly as high-speed directional (Fig. 1c). These state predictions correlated to dive patterns during a 24-h period around the time of exposure, with the high-speed directional state often being associated with repeated diving to intermediate depths (Fig. 3a). Four of the six whales initiated a long (1.2-2.2 h) and deep (992-1552 m) dive during exposure (Fig. 3a). The only deep dive for which higher-resolution depth measurements were collected by the satellite tag had a shallow ascent (ID161591).

Echolocation clicks of northern bottlenose whales were detected in the PAM recording during exposure, and these detections were followed by a click-absent period that started when the sonar was still transmitting (Fig. S3b). The duration of this period was 13h55min (13h46min according to the auditor). This observation was a statistical outlier (at 0.05 level) compared with the durations of pre-exposure click-absent periods (Fig. S3c), suggesting that the experimental exposure caused whale groups near the moored acoustic recorder location (25 km from the source) to stop echolocating and/or move out of the area. The received SPL for these groups at the start of the click-absent period was 95 dB re 1 µPa (90% confidence interval from 80-103 dB re 1 µPa; Table S2).

Long-term averaged spectrograms

To give an indication of the pristineness of the Jan Mayen area, the presence of active sonar was detected using long-term averaged spectrograms (LTSA) of acoustic data that were recorded on the bottom-moored acoustic recorders over a 2-year period (June 2015 – June 2017). LTSAs were constructed by computing for each frequency the maximum short-term PSD averaged over N=8192 samples (corresponding to ~0.06 s) within a 2.5-min period. These LTSAs were then manually inspected for presences of naval sonar, which were identified as instances where a significant acoustic energy was observed within a limited frequency band of 1-10 kHz. Sonar activities would have been easily detectable for SPLs exceeding the ambient levels by 10 dB, corresponding to roughly ~100 dB re 1 µPa for the average ambient noise conditions on the recorders. During the recording periods outside of June, the recorders were sampled at a duty cycle of 6% (2.5 min every 45 min). We could therefore not exclude that short duration exposures were present during the two-year period monitored. However, since sonar operations tend to continue over longer periods, it is likely that nearby sonars would have been picked up by this method.

Supplementary figures

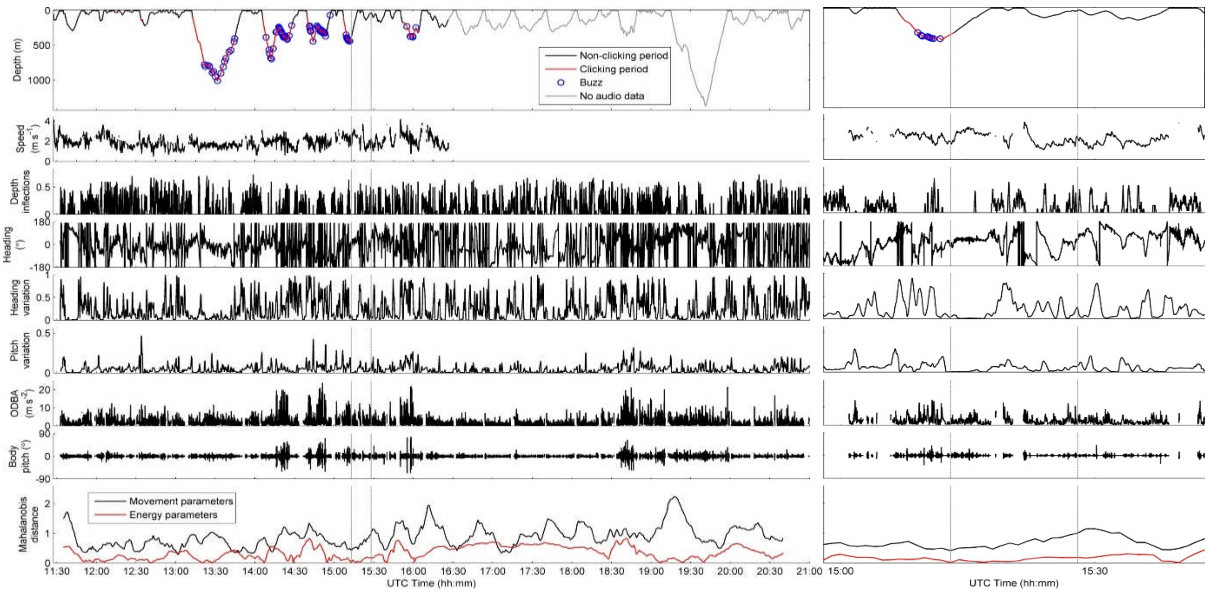

Figure S1: DTAG data from a northern bottlenose whale (ha15_171a), which underwent controlled exposure to naval sonar during experiment 2015-1. Grey vertical lines indicate the start and end of the exposure period.

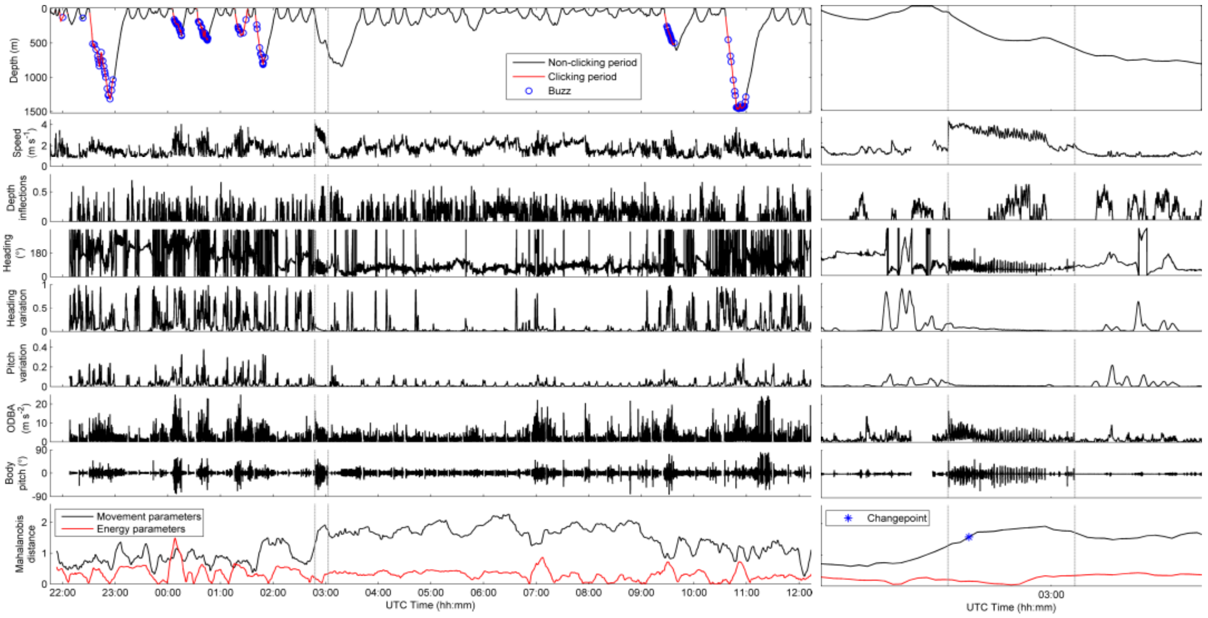

Figure S2: DTAG data from a northern bottlenose whale (ha15_179b), which underwent controlled exposure to naval sonar during experiment 2015-2. Grey vertical lines indicate the start and end of the exposure period.

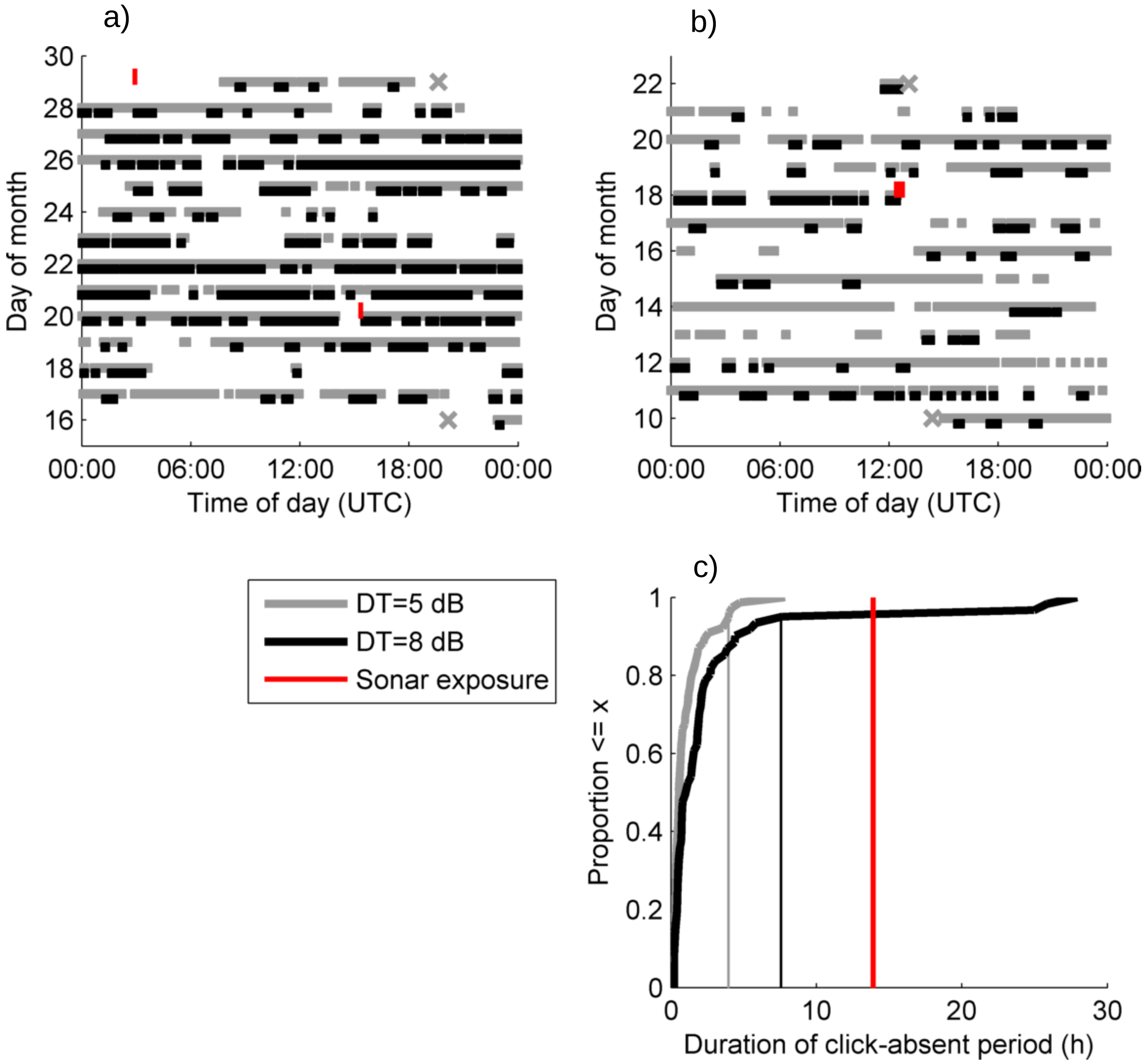

Figure S3: Northern bottlenose whale click-present periods (at two detector thresholds) measured in the PAM recordings during June a) 2015 and b) 2016, and timing of the experiments. Grey crosses mark the start and end of the recordings. c) Empirical cumulative distributions of pre-exposure click-absent period durations for 2016, the year in which a 14-h cessation in clicking (red line) was observed that started during the sonar exposure experiment (black and grey vertical lines indicate the 95th percentiles).

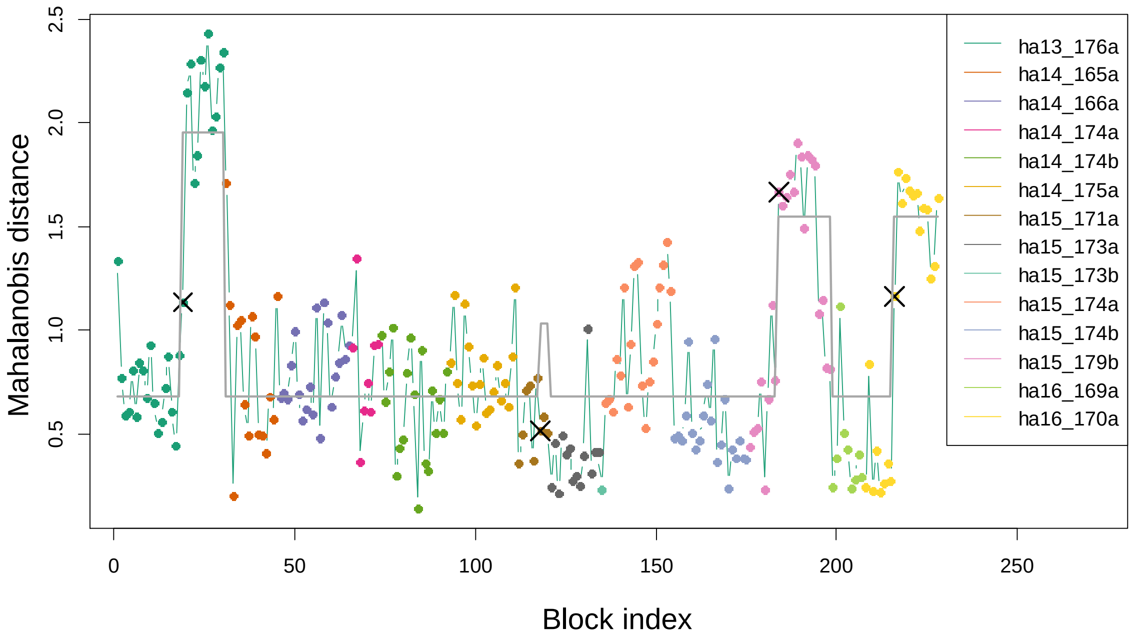

Figure S4: Observed values (coloured markers) and predictions from the selected response intensity model (grey line) as function of time block index, with the observations in chronological order. Each observation represents the average MD in the 35-min time block. Time blocks that correspond to the controlled exposures are indicated (black crosses).

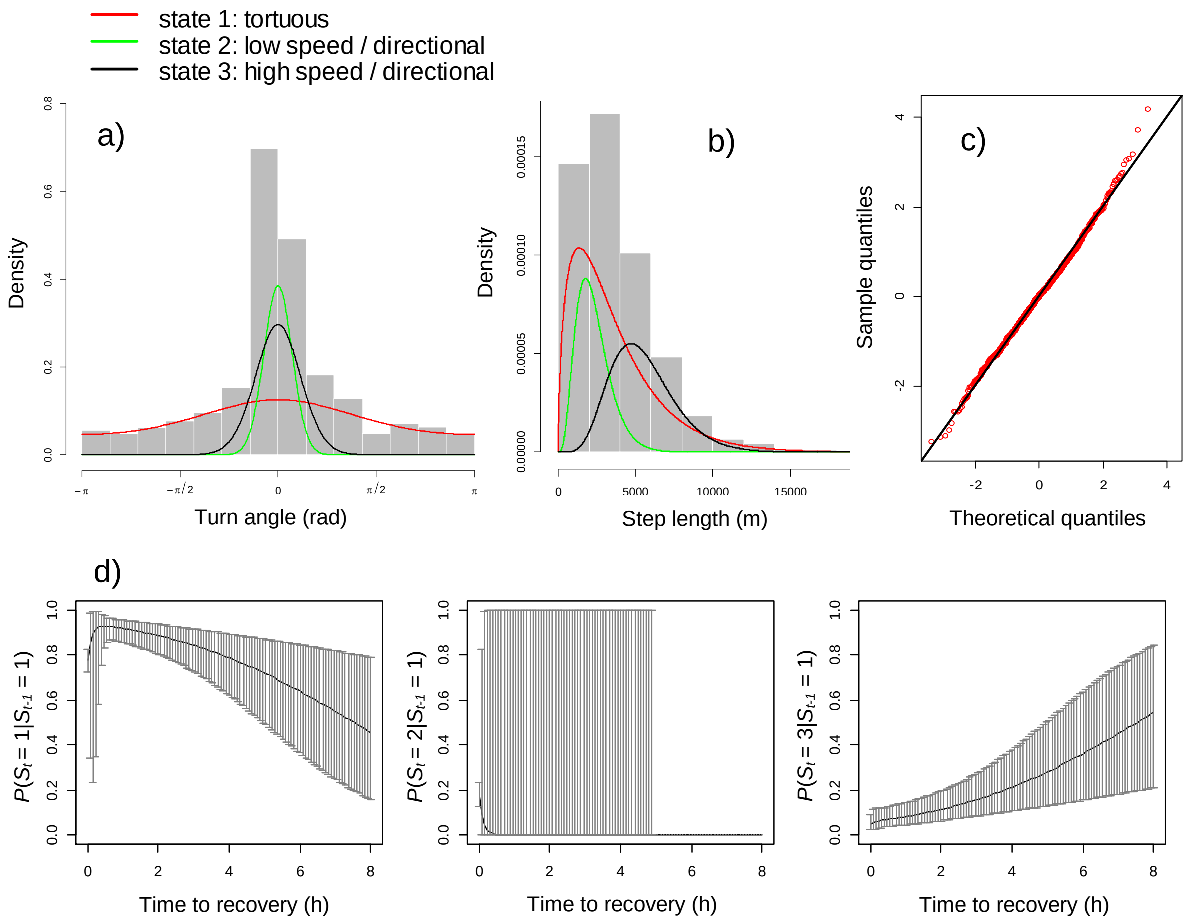

Figure S5: Results of the state-based modelling of satellite tag data of all individuals, with the estimated state-specific distributions for the a) turn angles and b) step lengths, a quantile-quantile plot of the pseudo-residuals for the step lengths, against the standard normal distribution, and d) the estimated probabilities for transitioning from state 1 to states 1, 2, and 3 as function of time to recovery. The maximum time to recovery (8 h) corresponded to the time-step of the controlled exposure. Error bars represent the 95% confidence intervals of the transition probability estimates.

Figures S6-S14 (below). Filtered track for each satellite-tagged whale. The colour-coding indicates the most likely sequence of states derived using the Viterbi algorithm. The blue arrow shows in which direction the animal was moving and the blue circle indicates the whale's location during the sonar exposure. The coordinates are UTM eastings and northings (zone 29W). Figures are shown in order of mean source distance during exposure.

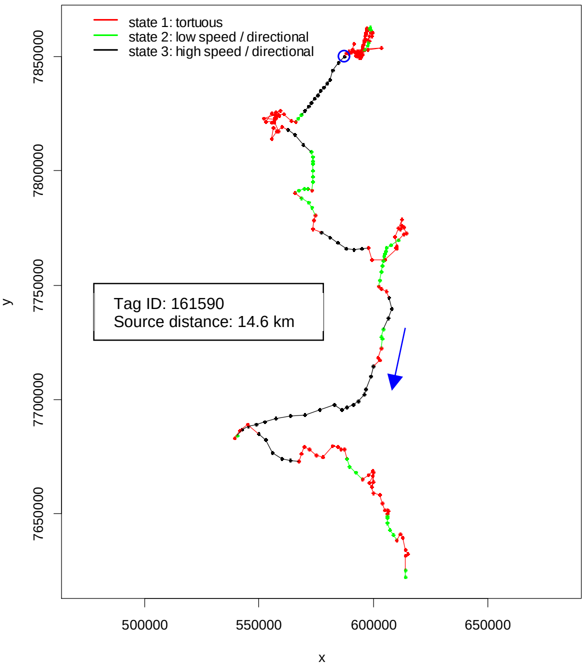

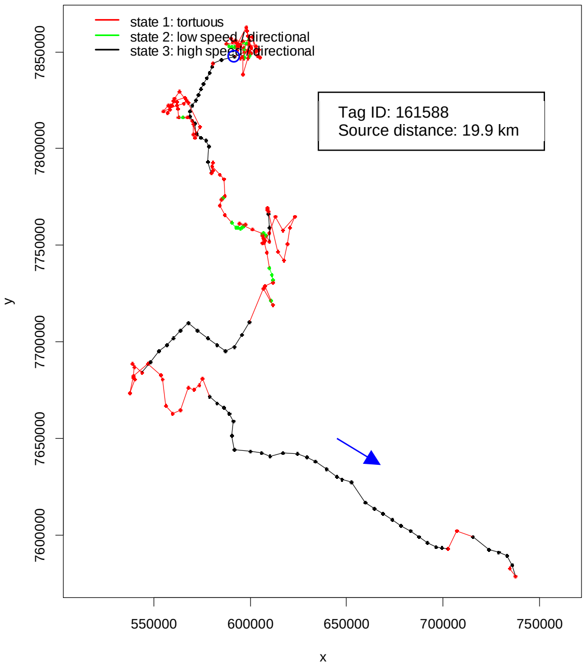

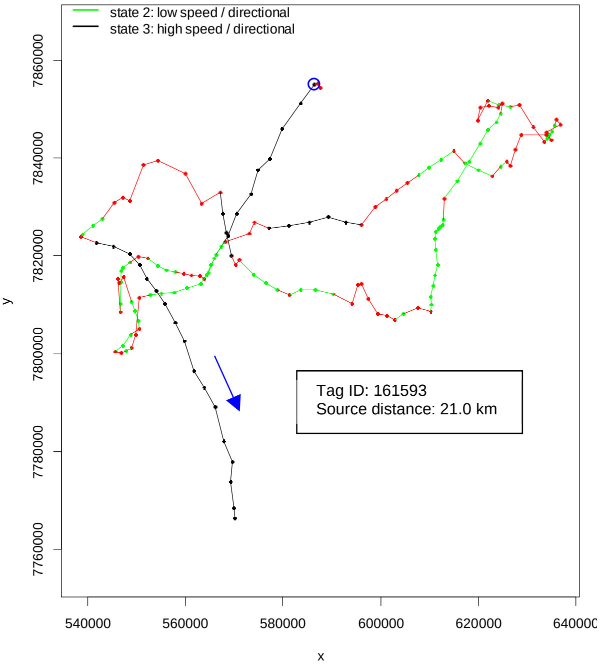

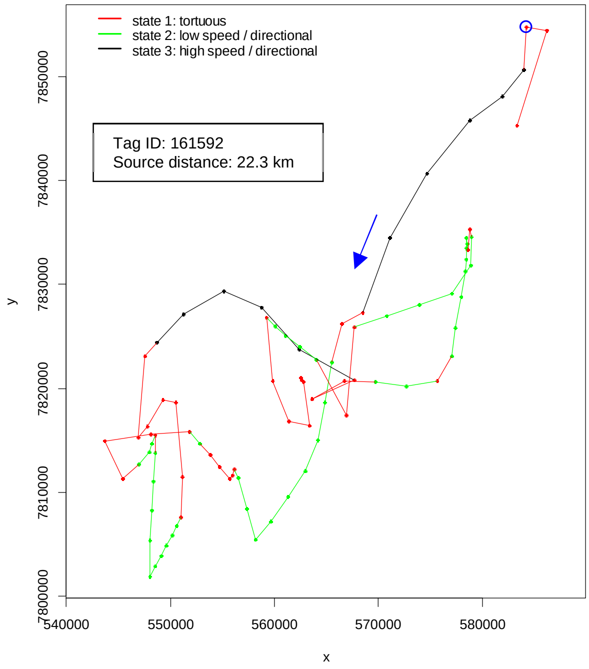

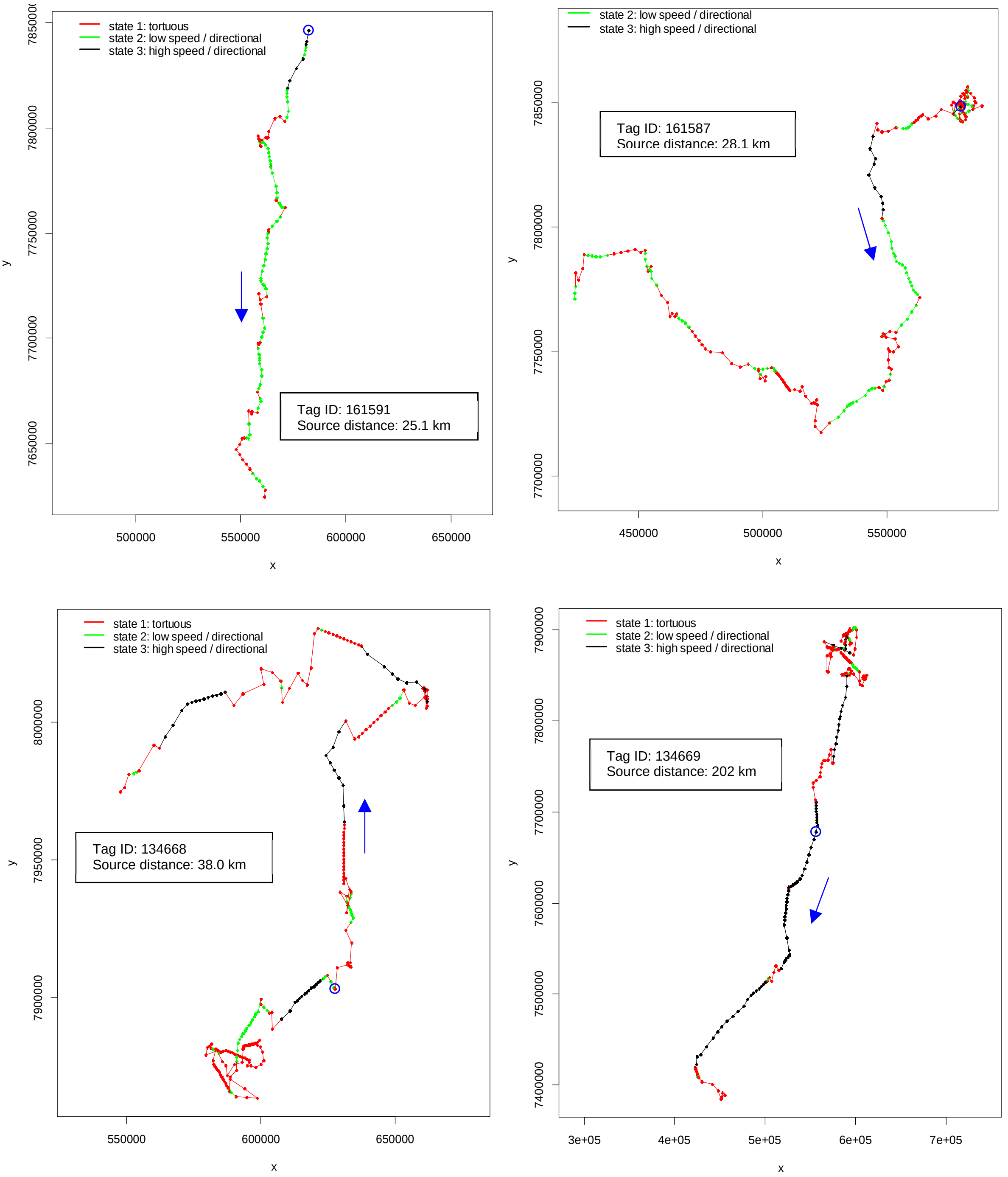
state 1: tortuous
state 2: low speed / directional
state 3: high speed / directional
Tag ID: 161591
Source distance: 25.1 km
500000
550000
600000
650000
7650000
7700000
7750000
7800000
x
y
Tag ID: 161587
Source distance: 28.1 km
450000
500000
550000
7700000
7750000
7800000
7850000
Tag ID: 134668
Source distance: 38.0 km
550000
600000
650000
7900000
7950000
8000000
Tag ID: 134669
Source distance: 202 km
3e+05
4e+05
5e+05
6e+05
7e+05
7400000
7500000
7600000
7700000
7800000
7900000

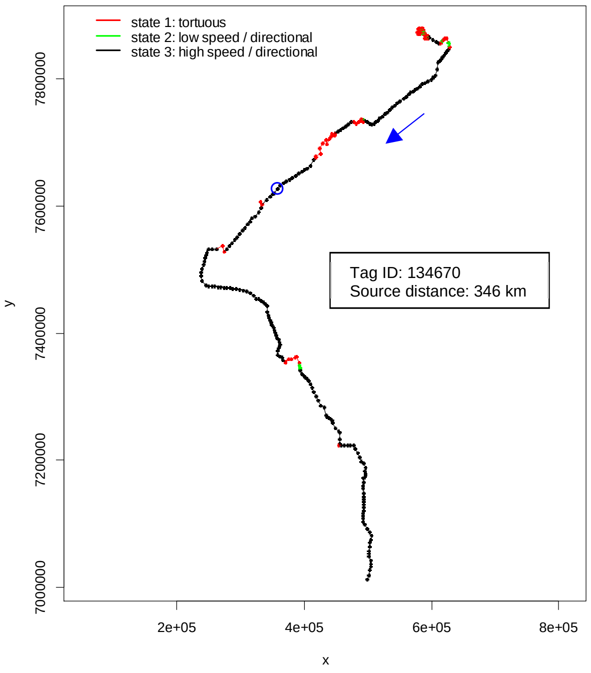
state 1: tortuous
state 2: low speed / directional
state 3: high speed / directional
Tag ID: 134670
Source distance: 346 km
7000000
7200000
7400000
7600000
7800000
y
2e+05
4e+05
6e+05
8e+05
x

Supplementary tables

Table S1. Data sets recorded with animal-borne tags and bottom-moored acoustic recorders that were analysed for behavioural effects of the controlled sonar exposure experiments.

Data set	Data type	Device	Deployment time		Deployment location		Experiment
			Start (UTC)	Duration	Latitude (deg)	Longitude (deg)	
ha15_171a	High-resolution tag	DTAG2	20-Jun-2015 11:27	9.6h	70.8870	-6.0814	2015-1
ha15_179b	High-resolution tag	DTAG3	28-Jun-2015 21:45	14.5h	71.0546	-6.2660	2015-2
ha16_170a	High-resolution tag	Mixed-tag /w DTAG3 core, Fastloc-GPS and SPOT	18-Jun-2016 07:06	13.1h	70.7378	-6.4865	2016-1
134670	Satellite tag	SPLASH10-292B	22-Jun-2015 14:45	41d 21h	70.9859	-6.6736	2015-2
134669	Satellite tag	SPLASH10-292B	22-Jun-2015 15:59	8d 16h	70.9565	-6.7595	2015-2
134668	Satellite tag	SPLASH10-292A	23-Jun-2015 20:42	26d 19h	70.9857	-6.5652	2015-2
161587	Satellite tag	SPLASH10-292B	15-Jun-2016 09:32	35d 7h	70.7449	-6.5316	2016-1
161588	Satellite tag	SPLASH10-292B	15-Jun-2016 22:35	35d 3h	70.7354	-6.5663	2016-1
161590	Satellite tag	SPLASH10-292B	16-Jun-2016 04:30	33d 1h	70.7618	-6.5392	2016-1
161591	Satellite tag	SPLASH10-292B	18-Jun-2016 10:30	5d 23h	70.7588	-6.5075	2016-1
161592	Satellite tag	SPLASH10-292B	18-Jun-2016 08:13	4d 20h	70.7407	-6.4940	2016-1
161593	Satellite tag	SPLASH10-292B	18-Jun-2016 08:15	38d 1h	70.7407	-6.4934	2016-1
JM1	Moored recorder	Loggerhead DSG-ST	16-Jun-2015 22:40	13d 0h	71.0320	-7.0286	2015-1, 2015-2
JM5	Moored recorder	Loggerhead DSG-ST	10-Jun-2016 14:57	11d 23h	70.9254	-6.5607	2016-1

Table S2. Received levels and source-whale distances during the sonar exposures for tagged whales and whales near the bottom-moored recorder. Received levels prior to response onset and source distances at response onset are reported if it was possible to identify the exact onset time. The 5th and 95th percentiles for received level predictions that were based on acoustic propagation modelling (satellite tags) or extrapolated from one SPL measurement (DTAG in experiment 2015-1; see footnote) are shown in parentheses. Response was coded as 'At' for attraction, 'Av' for avoidance, '-' for unable to assess, or '0' for no response. Behavioural thresholds for attraction and avoidance observed by Miller et al. (2015) are provided as a reference.

Experiment	Data type	Data set	Response	Received SPL (dB re 1 μPa)		Received SELcum (dB re 1 μPa^2 s)		Source-whale distance (km)	
				response onset	maximum	response onset	maximum	response onset	min/max range
2013	DTAG	ha13_176a	At	98	151	100	162	5.2	4.4-7.7
			Av	130	151	128	162	4.5	4.4-7.7
2015-1	DTAG	ha15_171a	At	66 (56, 76)*	99	66 (56, 76)*	104 (94, 114)*	0.6	0.02-0.6
	Acoustic recorder	JM1	0		**				36.9-37.1
2015-2	DTAG	ha15_179b	Av	127	128	125	126	0.8	0.8-3.4
	Satellite tag	134668	0		82 (78, 88)		103 (103, 104)		37.8-38.2
	Satellite tag	134669	-		71^^		88^^		201-202
	Satellite tag	134670	0		62^^		79^^		345-346
	Acoustic recorder	JM1	-		86 (81, 91)				25.5-25.6
2016-1	DTAG	ha16_170a	Av	117	128	124	134	16.7	16.6-18.8
	Satellite tag	161587	Av		121 (115, 127)		133 (132, 134)		27.1-29.0
	Satellite tag	161588	Av		124 (116, 137)		138 (137, 141)		19.0-20.7
	Satellite tag	161590	Av		126 (116, 138)		139 (137, 144)		12.8-16.3
	Satellite tag	161591	Av		121 (108, 129)		133 (131, 135)		25.0-25.2
	Satellite tag	161592	Av		120 (114, 134)		136 (133, 139)		22.2-22.4
	Satellite tag	161593	Av		122 (116, 137)		136 (133, 139)		20.2-21.7
	Acoustic recorder	JM5	Av***	95 (80, 103)^	120 (112, 127)				24.3-25.0

* Only one sonar pulse, received at minimum distance, was detected in the audio recording. Therefore, the received levels were estimated by assuming spherical spreading and applying a correction for the difference in source-whale distance ($20 * \log_{10}(15.2 / 632.5)$) with the measured SPL. This correction factor was assumed to be accurate (90% confidence interval) within plus/minus a factor of ~3 based upon the typical position uncertainty in this type of movement tracks and the fact that the whale was sighted <1 min before it was at the minimum distance to the source.
^ Response onset was taken as the time when last faint click was detected in the recording.
** Received levels omitted as they were far below the expected ambient noise levels because of the low SL.
^^ Received levels are rough estimates based upon spherical spreading with absorption loss at 1 kHz (0.036 * distance(km); Richardson et al., 1995).
*** Cessation of sound production that was judged to be potentially associated with avoidance to the sonar exposure. Response onset corresponded with the start of 14-h click-absent period in the PAM recording.

Table S3. AICs for response intensity models fitted to DTAG data.

AIC	ΔAIC	Parameters	Model description
97.7	0	ω, β_0, β_1	Effect of received level
99.4	1.6	$\omega, \beta_0, \beta_1, \beta_2$	Effects of received level and time since exposure
99.7	2.0	$\omega, \beta_0, \beta_1, \beta_3$	Effects of received level and distance
101.4	3.6	$\omega, \beta_0, \beta_1, \beta_2, \beta_3$	Full model
156.7	58.9	$\omega, \beta_0, \gamma, \beta_2$	Effect of time since exposure
157.3	59.6	$\omega, \beta_0, \gamma, \beta_3$	Effect of distance
158.7	60.9	$\omega, \beta_0, \gamma, \beta_2, \beta_3$	Effects of time since exposure and distance
260.6	162.8	ω, β_0	Baseline model

Table S4. AICs for hidden Markov models fitted to satellite tag data of all individuals.

AIC	ΔAIC	Covariate	Model description
29135.5	0	time to recovery × SPLmax	Received level-dependent effect of sonar on transitioning from state 1 to states 1, 2, and 3
29137.0	1.5	time to recovery	Effect of sonar on transitioning from state 1 to states 1, 2, and 3
29145.5	9.9	-	Baseline model
29146.2	10.6	time to recovery × distance	Distance-dependent effect of sonar on transitioning from state 1 to states 1, 2, and 3

Table S5. ΔAICs for hidden Markov models fitted to satellite tag data of reduced sets of individuals. Based on visual surface observation and a comparison of the horizontal movement and diving patterns of the whales, we judged that ID161592 and ID161593 were probably associated with each other and ID161588 and ID161590 were possibly associated with each other, during the 8-h period following the sonar exposure.

	Excl. 161592	Excl. 161593	Excl. 161588 & 161592	Excl. 161590 & 161592	Excl. 161588 & 161593	Excl. 161590 & 161593
	ΔAIC	ΔAIC	ΔAIC	ΔAIC	ΔAIC	ΔAIC
RL-dependent effect of sonar	0	0	0	0	0.0	0.1
Effect of sonar	0.8	0.3	0.3	0.9	0	0
Baseline model	5.8	2.7	2.1	3.2	1.2	5.6
Distance-dependent effect of sonar	5.8	3.6	2.4	5.2	0.7	2.9

Table S6. Results of the expert-identification analysis. A 'severity score' according to the 10-point scale in Southall et al. (2017) is provided for each identified response.

Experiment / tag id	Exposure type	Exposure duration	Justification	Onset of response: Description	Onset of response: UTC time	End of response: Description	End of response: UTC time	Response duration	Confidence	Received SPLmax	Received SELcum	Source distance	avoidance	orientation	locomotion	dive profile	foraging	resting	vocal behav.	group dist.	mother-calf	aggression
2015-1 / ha15_171a	Close	15	Moderate change in locomotion. Attraction based on track. The animal approached the source and started milling around the source.	At small right turn early on during exposure	20-06-2015 15:13:16 (+00:00:16)	Until end of exposure	20-06-2015 15:28:00	15 min	High	66.3	66.3	0.633			4							
2015-1 / ha15_171a	Close	15	Moderate cessation of feeding	When animal stops echolocating	20-06-2015 15:13:22 (+00:00:22)	When animal starts echolocating	20-06-2015 15:52:03	40 min	High	66.5	69.5	0.618					6					
2015-1 / ha15_171a	Close	15	Change in vocal behavior	When animal stops echolocating	20-06-2015 15:13:22 (+00:00:22)	When animal starts echolocating	20-06-2015 15:52:03	40 min	High	66.5	69.5	0.618							4			
2015-1 / ha15_171a	Close	15	No change in dive behavior. An unusual long and shallow dive starting midway the exposure, but similar dives seen during baseline.													0						
2015-2 / ha15_179b	Close	15	Prolonged avoidance. Animal turned away from the source just before exposure, but had a dramatic increase in speed (+startle response) at the start. Swim speed decreased near end of exposure but animal continued to swim directly away for hours	At increase in swim speed	29-06-2015 02:48:00 (+00:00:00)	When animal starts echolocating	29-06-2015 09:26:37	6.5 h	High	127.2	124.8	0.814	7									
2015-2 / ha15_179b	Close	15	Prolonged cessation of feeding. Animal makes a deep dive without echolocating	When animal reaches foraging depth without clicking (based on previous foraging dive)	29-06-2015 02:48:00 (+00:00:00)	When animal starts echolocating	29-06-2015 09:26:37	6.5 h	High	127.2	124.8	0.814					7					
2015-2 / ha15_179b	Close	15	Extended cessation of vocal behavior	Same as above	29-06-2015 02:48:00 (+00:00:00)	When animal starts echolocating	29-06-2015 09:26:37	6.5 h	High	127.2	124.8	0.814							6			
2015-2 / ha15_179b	Close	15	Prolonged change in the dive profile, with a deep avoidance dive at start of sonar and then a series of traveling dives with max depths of ~200m	At wiggle at start of exposure	29-06-2015 02:48:00 (+00:00:00)	After the 4 dives to 200m depth	29-06-2015 04:50:50	2 h	High	127.2	124.8	0.814				5						
2015-2 / ha15_179b	Close	15	Brief orientation response	At wiggle at about 10min into the exposure	29-06-2015 02:58:56 (+00:10:56)	Immediate		-	High	128.4	126.2	2.89		1								
2016-1 / ha16_170a	Distant	35	Prolonged cessation of feeding	When the animal reaches foraging depth without clicking (based on previous foraging dive)	18-06-2016 12:21:19 (+00:05:19)	Until end of the record	18-06-2016 20:14:30	>8 h	High	74.9	64.2	16.9					7					
2016-1 / ha16_170a	Distant	35	Minor change in locomotion, does 2 turns towards the source before the avoidance, possible orientation behaviour.	At start of first change in heading	18-06-2016 12:22:03 (+00:06:03)	Until start of avoidance	18-06-2016 12:36:26	14 min	High	76.5	75.0	16.8			3							
2016-1 / ha16_170a	Distant	35	Extended change in dive behavior. No more deep and shallow diving. All intermediate dives	At the start of the ascent of the first dive	18-06-2016 12:22:05 (+00:06:05)	Until end of the record	18-06-2016 20:14:30	>8 h	High	76.5	75.0	16.8				5						
2016-1 / ha16_170a	Distant	35	Extended cessation of vocal behavior	When the animal stops clicking	18-06-2016 12:30:15 (+00:14:15)	Until end of the record	18-06-2016 20:14:30	>7.5 h	High	105.6	104.8	16.6							6			
2016-1 / ha16_170a	Distant	35	Long term avoidance of area. Increase in swim speed, and then becomes directional. Satelite data and buoy data indicate avoidance responses were more than just prolonged.	At increase in swim speed and more directional movement, roughly halfway into the exposure	18-06-2016 12:36:26 (+00:20:26)	Until end of the record, and beyond based on sat. data	18-06-2016 20:14:30	>7.5 h	High	117.4	124.3	16.6	8									

References

Andrews RD, Pitman RL, Ballance LT. 2008 Satellite tracking reveals distinct movement patterns for Type B and Type C killer whales in the southern Ross Sea, Antarctica. Polar Biol. 31, 1461–1468. (doi:10.1007/s00300-008-0487-z)

Johnson MP, Tyack PL. 2003 A digital acoustic recording tag for measuring the response of wild marine mammals to sound. IEEE J. Ocean. Eng. 28, 3–12. (doi:10.1109/Joe.2002.808212)

Miller PJO et al. 2012 The severity of behavioral changes observed during experimental exposures of killer (*Orcinus orca*), long-finned pilot (*Globicephala melas*), and sperm (*Physeter macrocephalus*) whales to naval sonar. Aquat. Mamm. 38, 362–401. (doi:10.1578/AM.38.4.2012.362)

Miller PJO et al. 2015 First indications that northern bottlenose whales are sensitive to behavioural disturbance from anthropogenic noise. R. Soc. Open Sci. 2, 140484. (doi:10.1098/rsos.140484)

Richardson WJ, Greene CR, Malme CI, Thomson DH. 1995 Marine Mammals and Noise. Academic Press, San Diego, CA.

Southall BL et al. 2007 Marine mammal noise exposure criteria: Initial scientific recommendations. Aquat. Mamm. 33, 411–521. (doi:10.1578/AM.33.4.2007.411)

Appendix I. Standardised plots used for expert-identification of responses

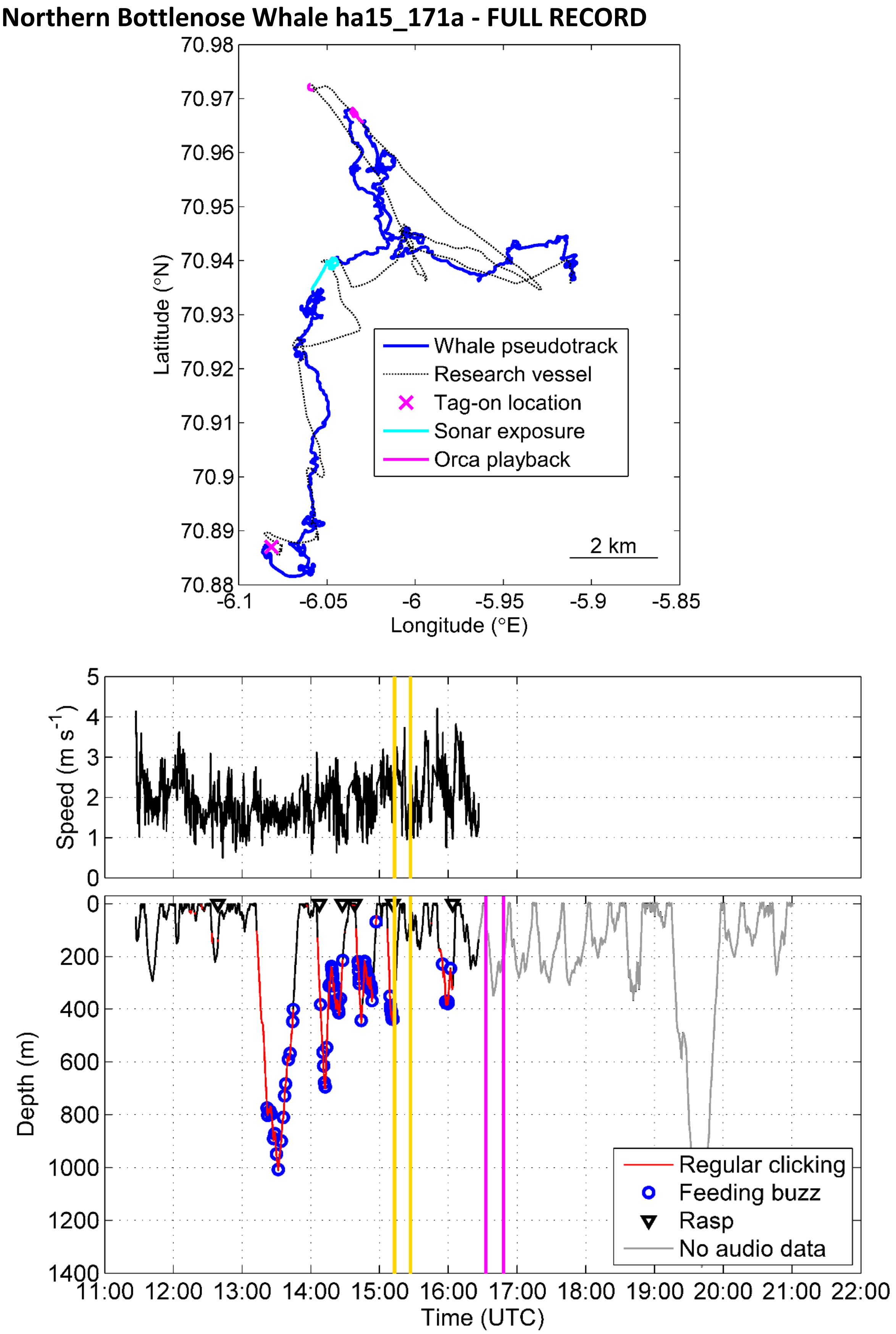

BASELINE

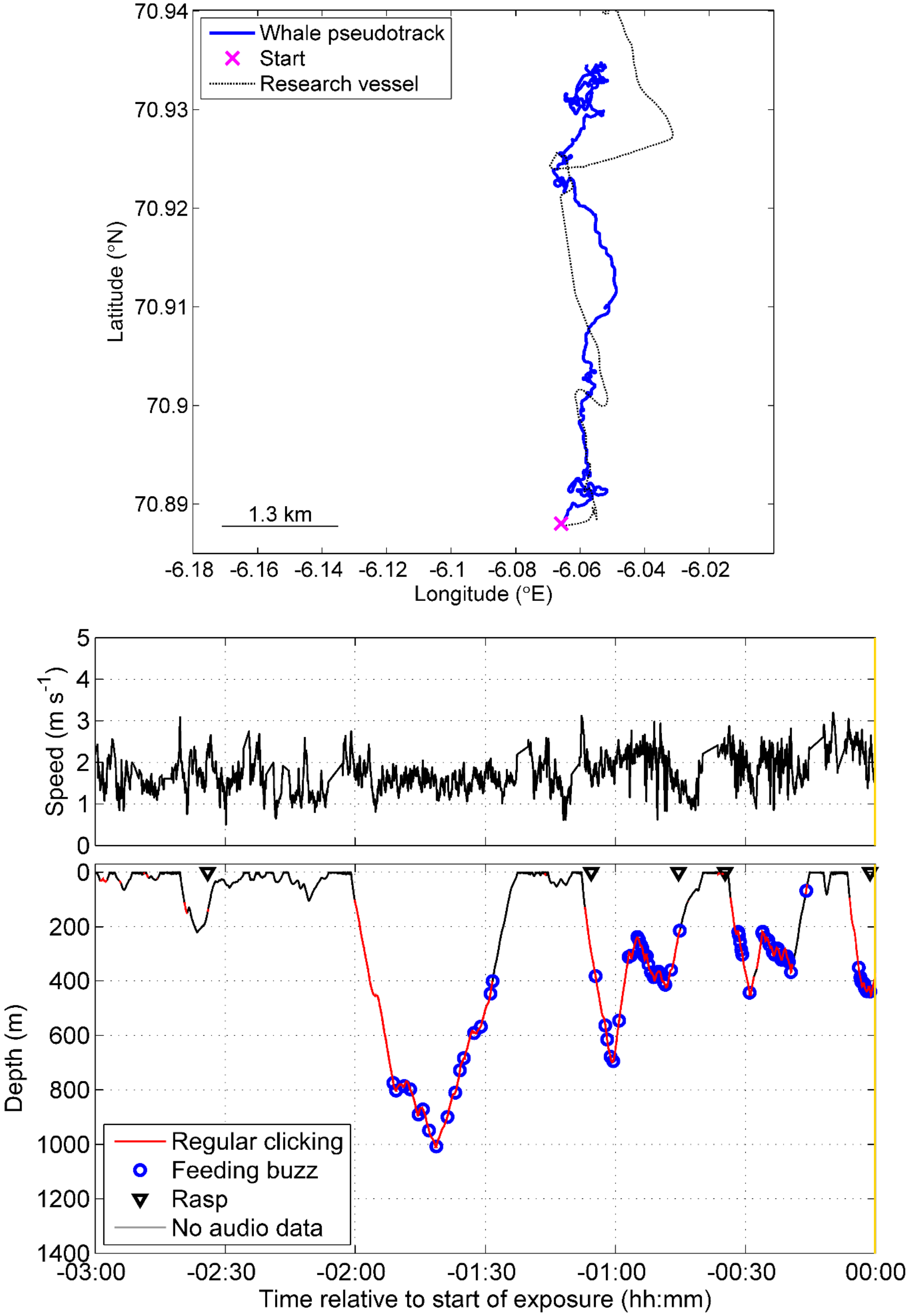

Whale pseudotrack
Start
Research vessel
1.3 km
Latitude (°N)
Longitude (°E)
70.94
70.93
70.92
70.91
70.9
70.89
-6.18
-6.16
-6.14
-6.12
-6.1
-6.08
-6.06
-6.04
-6.02
Speed (m s⁻¹)
Depth (m)
Regular clicking
Feeding buzz
Rasp
No audio data
Time relative to start of exposure (hh:mm)
-03:00
-02:30
-02:00
-01:30
-01:00
-00:30
00:00

SONAR

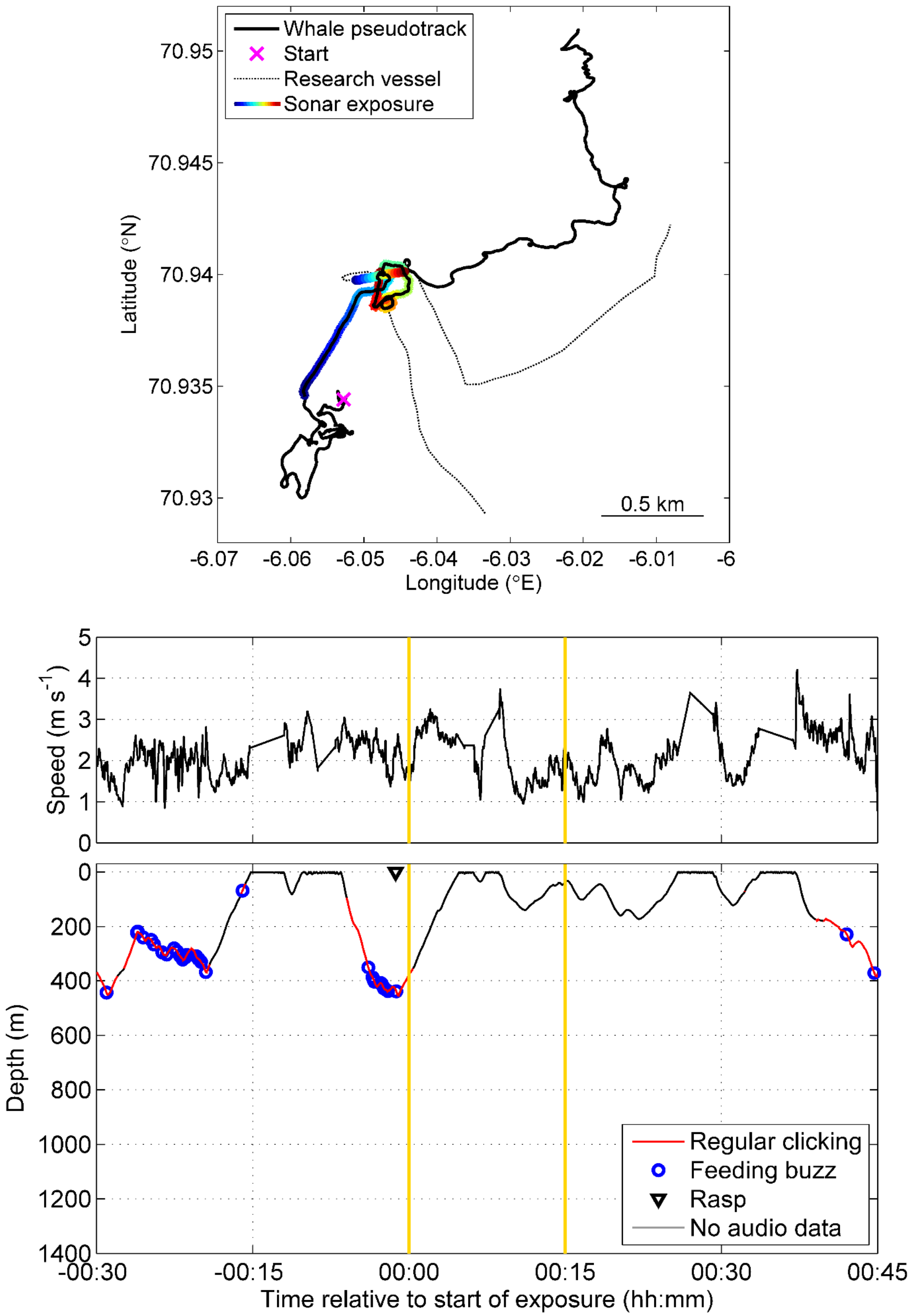

Whale pseudotrack
Start
Research vessel
Sonar exposure
70.95
70.945
70.94
70.935
70.93
Latitude (°N)
0.5 km
-6.07
-6.06
-6.05
-6.04
-6.03
-6.02
-6.01
-6
Longitude (°E)
Speed (m s^{-1})
5
4
3
2
1
0
Depth (m)
0
200
400
600
800
1000
1200
1400
Regular clicking
Feeding buzz
Rasp
No audio data
-00:30
-00:15
00:00
00:15
00:30
00:45
Time relative to start of exposure (hh:mm)

Northern Bottlenose Whale ha15_179b - FULL RECORD

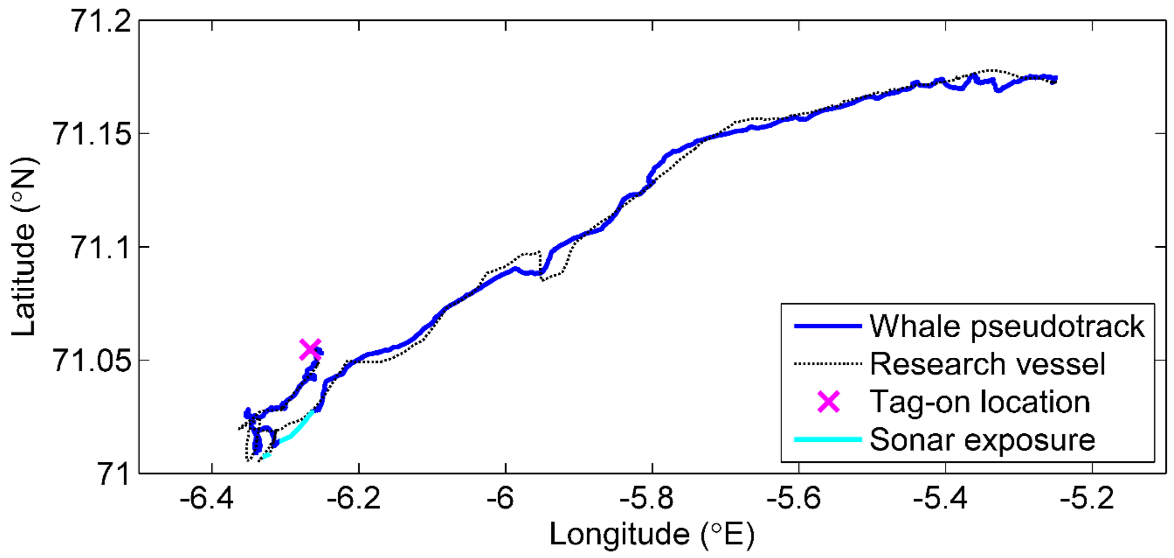

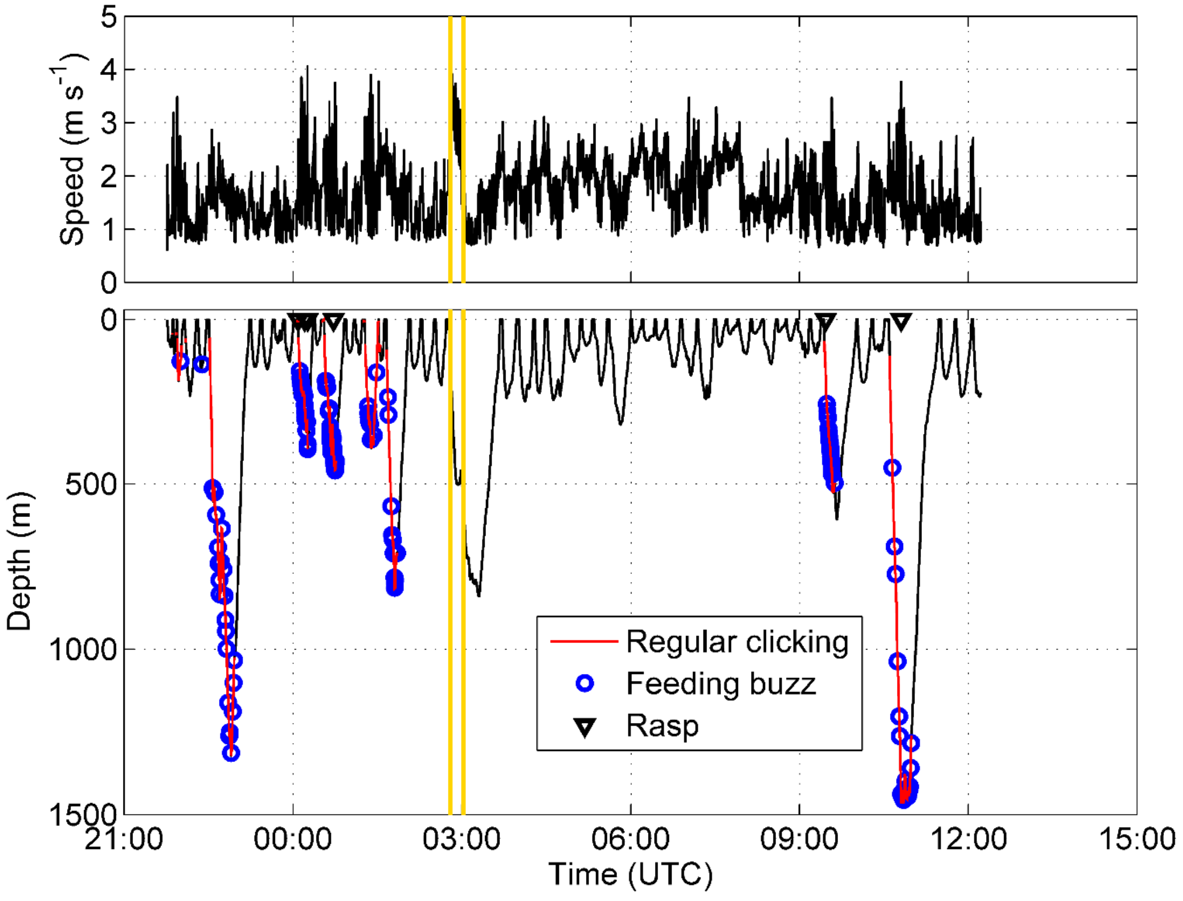

BASELINE

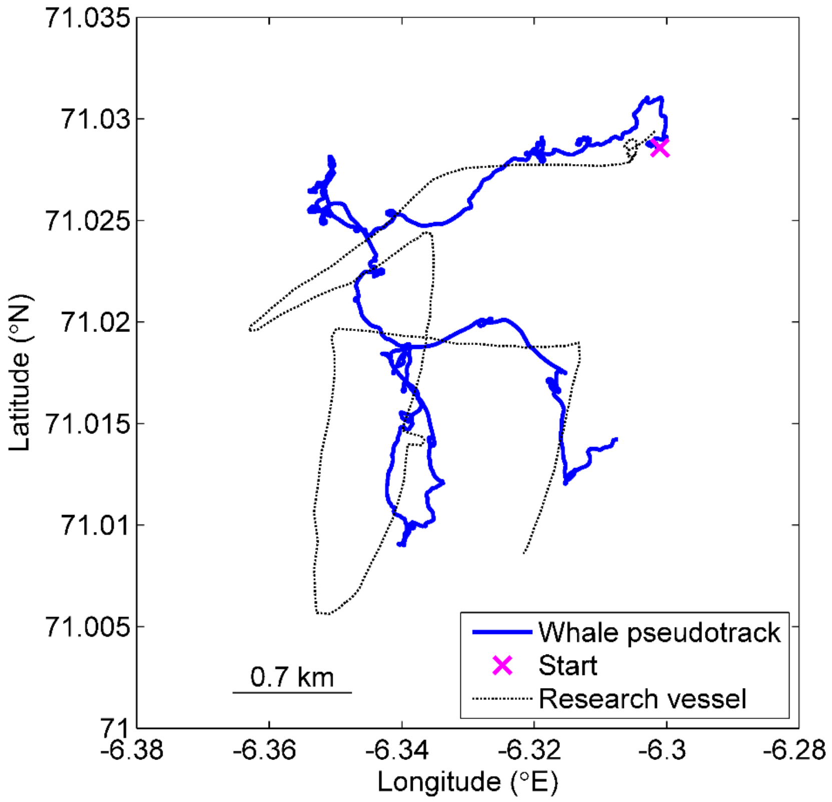

71.035
71.03
71.025
71.02
71.015
71.01
71.005
71
Latitude (°N)
-6.38
-6.36
-6.34
-6.32
-6.3
-6.28
Longitude (°E)
0.7 km
Whale pseudotrack
Start
Research vessel

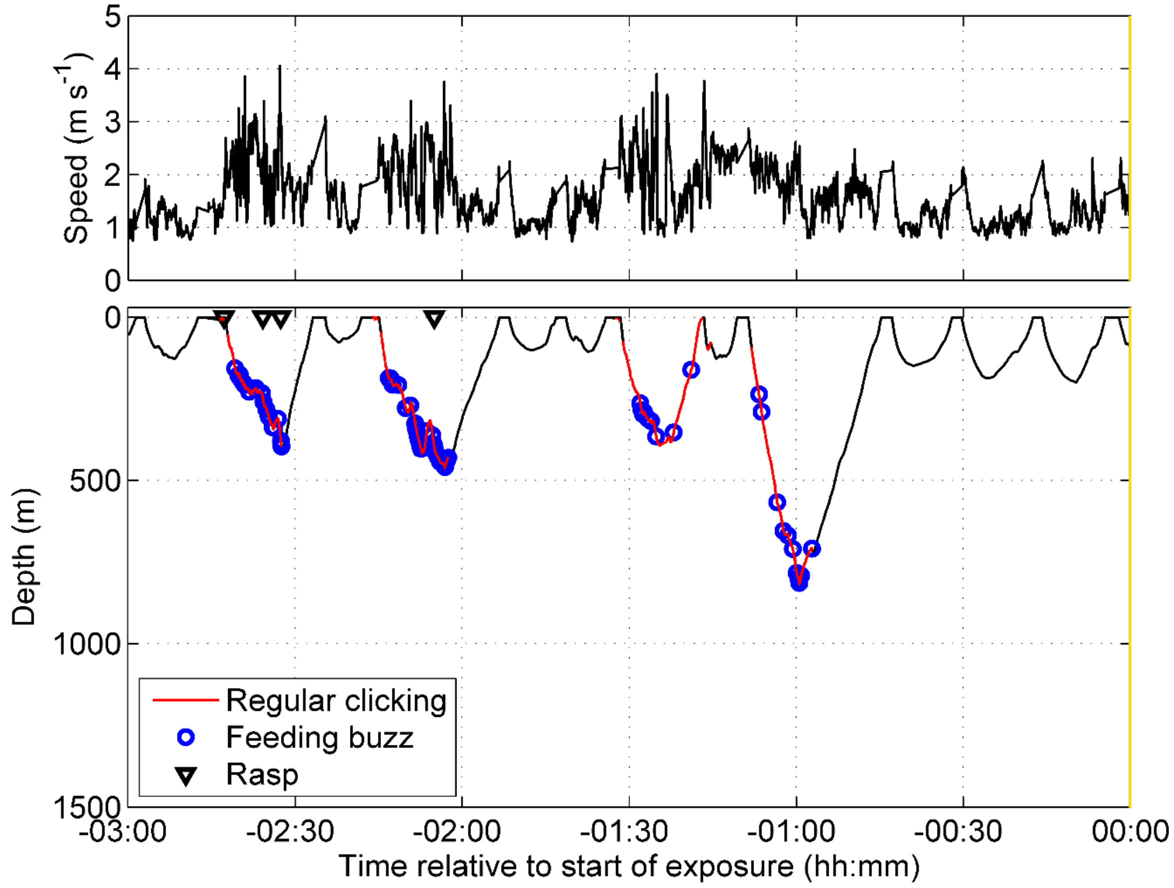

Speed (m s-1)
5
4
3
2
1
0
Depth (m)
500
1000
1500
Regular clicking
Feeding buzz
Rasp
-03:00
-02:30
-02:00
-01:30
-01:00
-00:30
00:00
Time relative to start of exposure (hh:mm)

SONAR

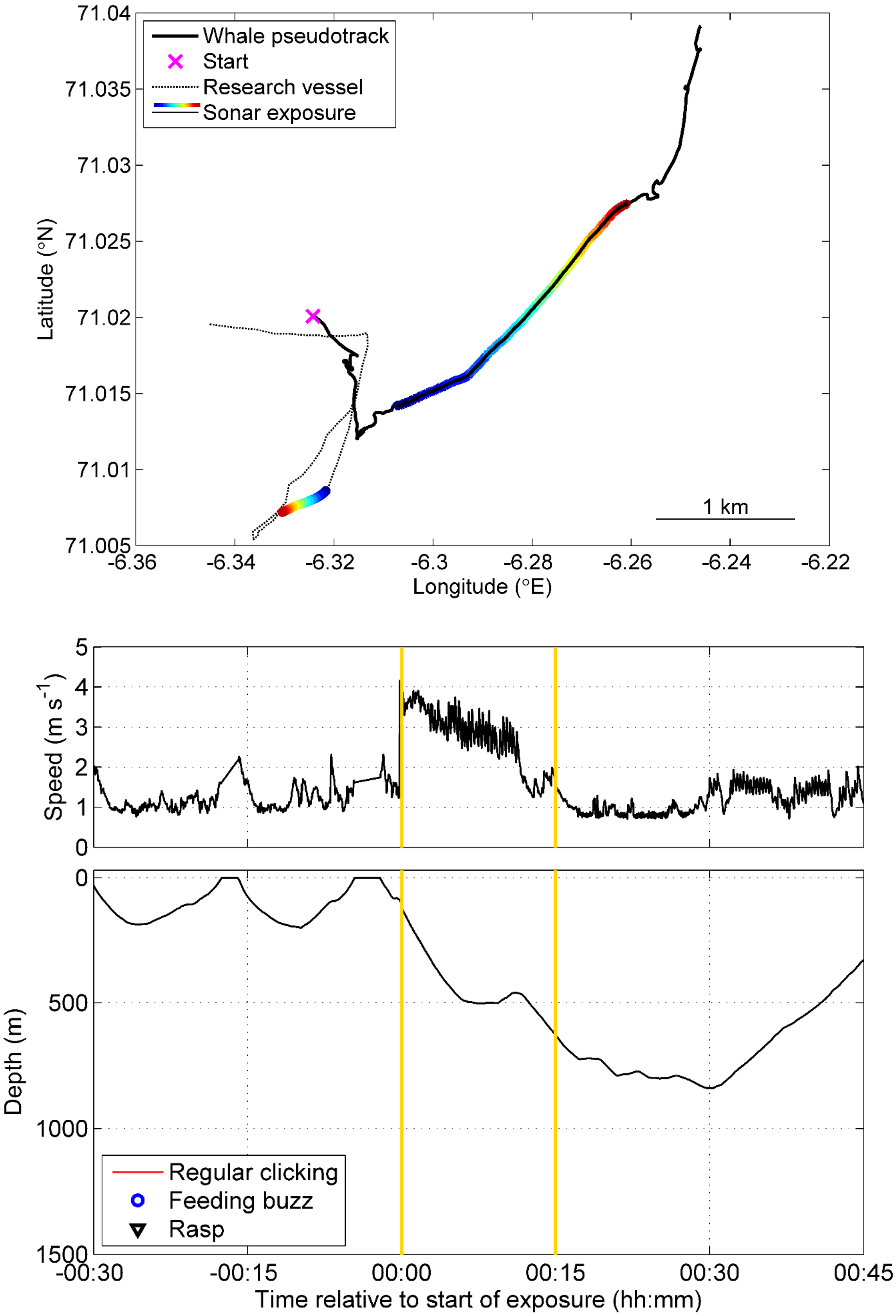
Whale pseudotrack
Start
Research vessel
Sonar exposure
1 km
Latitude (°N)
Longitude (°E)
71.04
71.035
71.03
71.025
71.02
71.015
71.01
71.005
-6.36
-6.34
-6.32
-6.3
-6.28
-6.26
-6.24
-6.22
Speed (m s-1)
Depth (m)
Regular clicking
Feeding buzz
Rasp
Time relative to start of exposure (hh:mm)
-00:30
-00:15
00:00
00:15
00:30
00:45

Northern Bottlenose Whale ha16_170a - FULL RECORD

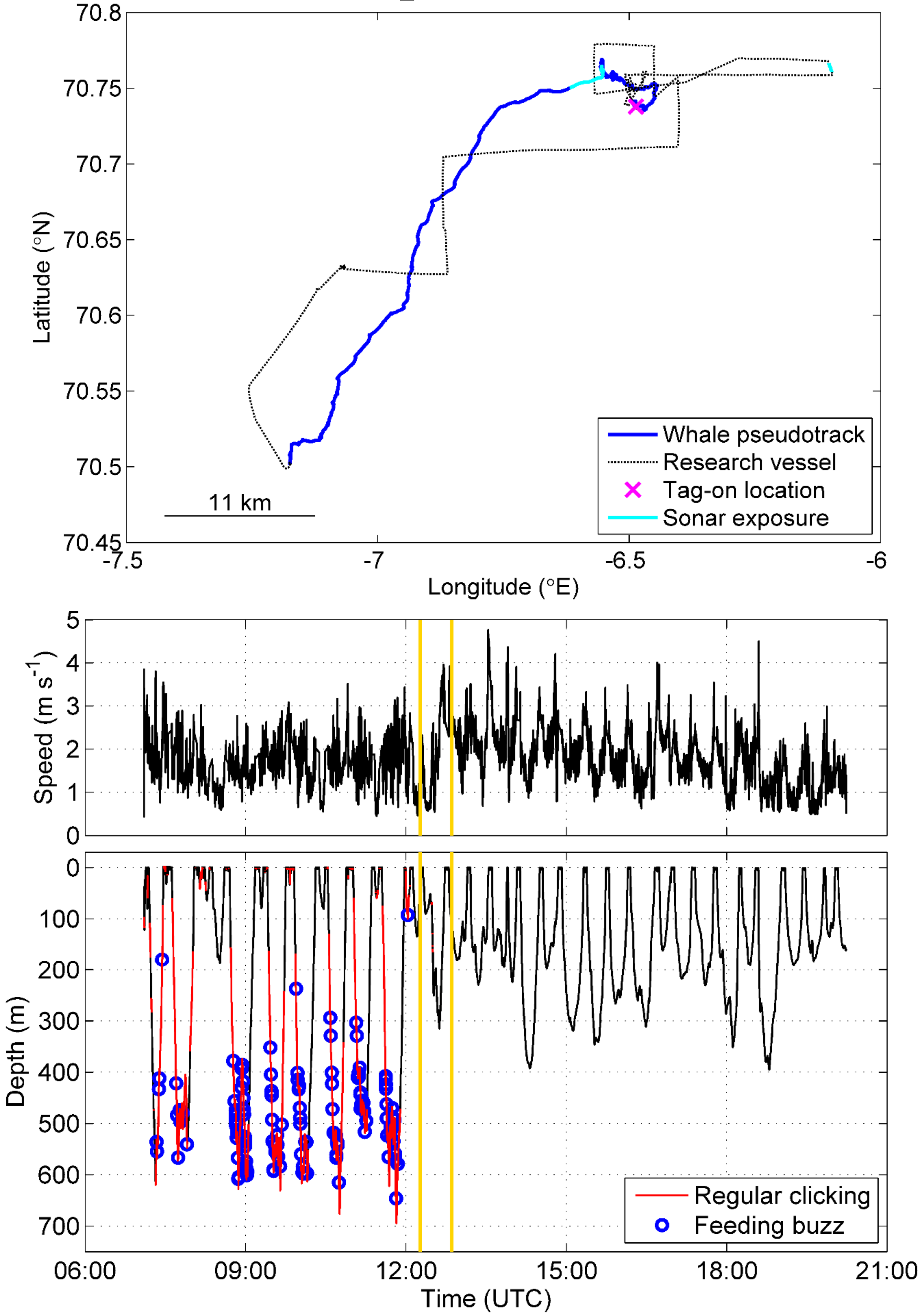

BASELINE

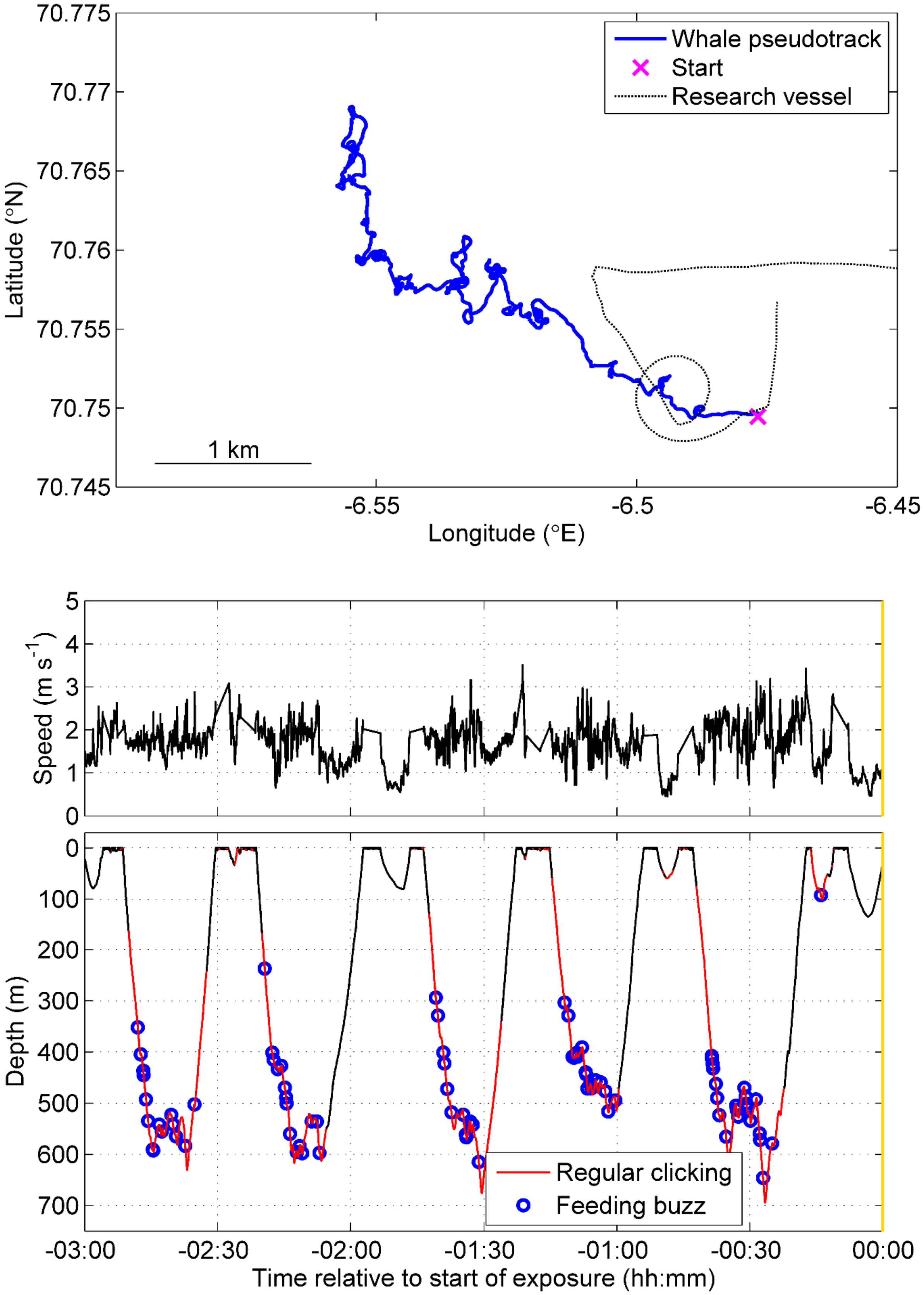

Whale pseudotrack
Start
Research vessel
1 km
Latitude (°N)
70.775
70.77
70.765
70.76
70.755
70.75
70.745
-6.55
-6.5
-6.45
Longitude (°E)
Speed (m s^{-1})
0
1
2
3
4
5
Depth (m)
0
100
200
300
400
500
600
700
Regular clicking
Feeding buzz
-03:00
-02:30
-02:00
-01:30
-01:00
-00:30
00:00
Time relative to start of exposure (hh:mm)

SONAR

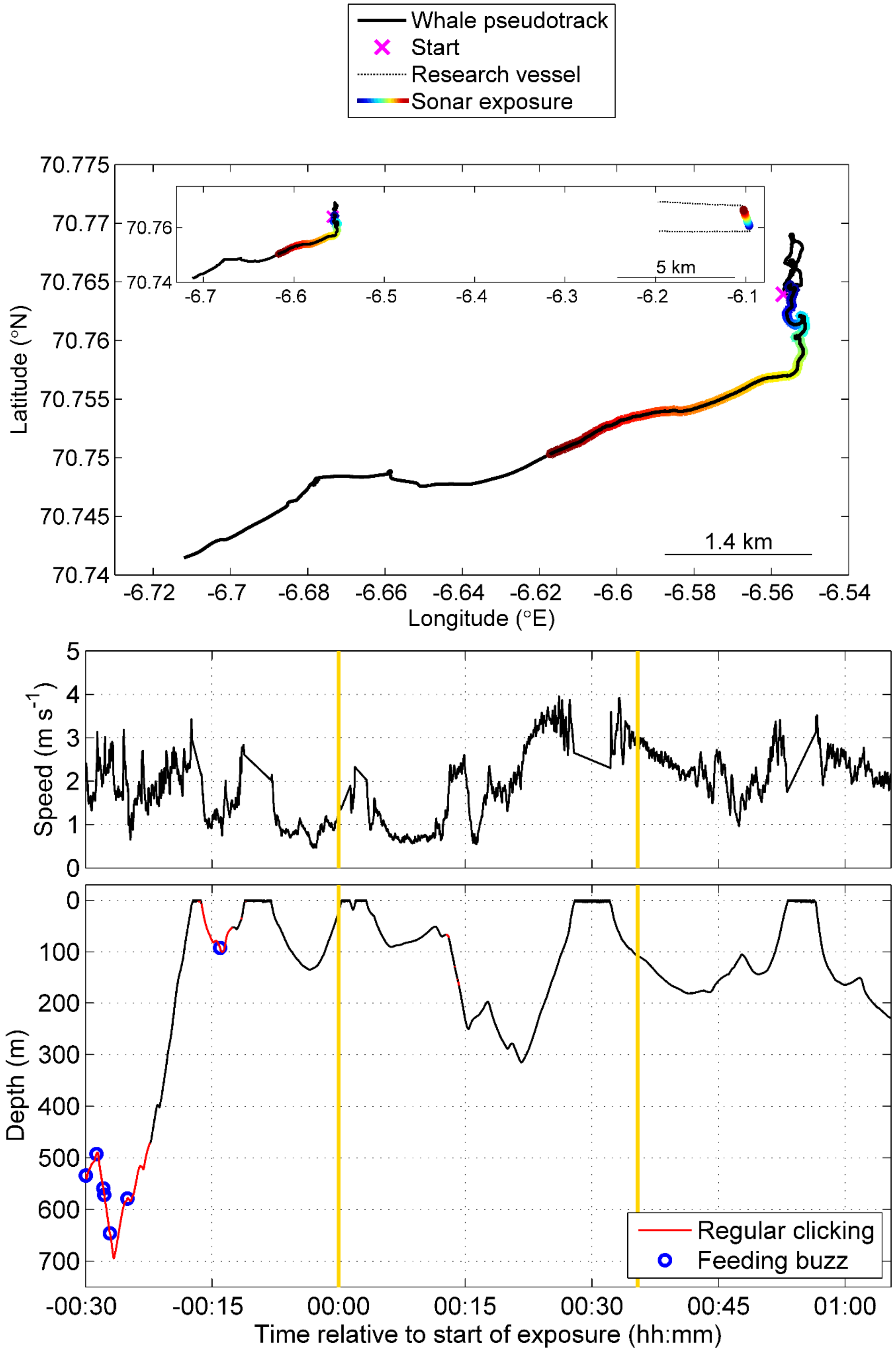
Whale pseudotrack
Start
Research vessel
Sonar exposure
70.775
70.77
70.765
70.76
70.755
70.75
70.745
70.74
Latitude (°N)
-6.72
-6.7
-6.68
-6.66
-6.64
-6.62
-6.6
-6.58
-6.56
-6.54
Longitude (°E)
1.4 km
70.76
70.74
-6.7
-6.6
-6.5
-6.4
-6.3
-6.2
-6.1
5 km
Speed (m s-1)
Depth (m)
Regular clicking
Feeding buzz
-00:30
-00:15
00:00
00:15
00:30
00:45
01:00
Time relative to start of exposure (hh:mm)
